# Integrated liver-secreted and plasma proteomics identify a predictive model that stratifies MASH

**DOI:** 10.1016/j.xcrm.2025.102085

**Published:** 2025-04-17

**Authors:** William De Nardo, Olivia Lee, Yazmin Johari, Jacqueline Bayliss, Marcus Pensa, Paula M. Miotto, Stacey N. Keenan, Andrew Ryan, Amber Rucinski, Tessa M. Svinos, Geraldine J. Ooi, Wendy A. Brown, William Kemp, Stuart K. Roberts, Benjamin L. Parker, Magdalene K. Montgomery, Mark Larance, Paul R. Burton, Matthew J. Watt

**Affiliations:** 1 Department of Anatomy and Physiology, School of Biomedical Sciences, Faculty of Medicine, Dentistry & Health Sciences, The University of Melbourne, Melbourne, VIC 3010, Australia; 2 Department of Surgery, School of Translational Medicine, Monash University, Melbourne, VIC 3004, Australia; 3 Bariatric Unit, Department of General Surgery, The Alfred Hospital, Melbourne, VIC 3004, Australia; 4 TissuPath, Mount Waverley, VIC 3149, Australia; 5 Department of Oncology, Bendigo Health, Bendigo, VIC 3550, Australia; 6 Department of General Surgery, Barwon Health, Geelong, VIC 3220, Australia; 7 Department of Gastroenterology, The Alfred Hospital and Monash University, Melbourne, VIC 3181, Australia; 8 Charles Perkins Centre and School of Medical Sciences, Faculty of Medicine and Health, University of Sydney, Sydney, NSW 2006, Australia

**Keywords:** hepatokine, MASL, MASLD, APASHA, proteome, APOF, AZGP1, small protein enrichment, protein secretion

## Abstract

Obesity is a major risk factor for metabolic-associated steatotic liver disease (MASLD), which can progress to metabolic-associated steatohepatitis (MASH). There are no validated non-invasive tests to stratify persons with obesity with a greater risk for MASH. Herein, we assess plasma and liver from 266 obese individuals spanning the MASLD spectrum. Ninety-six human livers were precision-cut, and mass spectrometry-based proteomics identifies 3,333 proteins in the liver-secretion medium, of which 107 are differentially secreted in MASH compared with no pathology. The plasma proteome is markedly remodeled in MASH but is not different between patients with steatosis and no pathology. The APASHA model, comprising plasma apolipoprotein F (APOF), proprotein convertase subtilisin/kexin type 9 (PCSK9), afamin (AFM), S100 calcium-binding protein A6 (S100A6), HbA1c, and zinc-alpha-2-glycoprotein (AZGP1), stratifies MASH (area under receiver operating characteristic [AUROC] = 0.88). Our investigations detail the evolution of liver-secreted and plasma proteins with MASLD progression, providing a rich resource defining human liver-secreted proteins and creating a predictive model to stratify patients with obesity at risk of MASH.

## Highlights

• Catalog of liver-secreted and plasma proteins with MASLD progression in humans
• Mass spectrometry analysis identifies remodeling of plasma proteins in human MASH
• Protein secretion from the liver is altered in human MASH
• The APASHA model effectively stratifies patients with obesity at risk of MASH

## Graphical abstract

**Figure:**
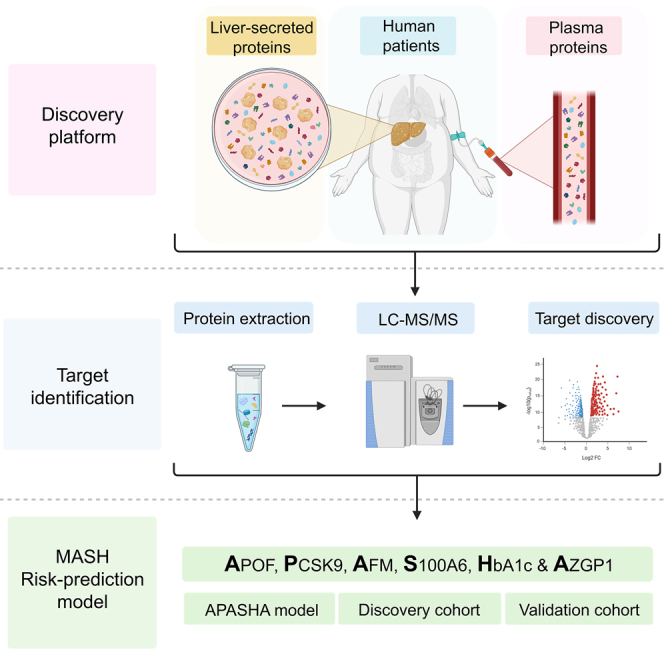

## Teaser

De Nardo et al. profile the plasma and liver-secreted proteome from obese individuals spanning the spectrum of MASLD. They identify biomarkers for MASH in persons with obesity and develop and validate a biologically plausible non-invasive risk prediction model to predict or exclude MASH.

## Introduction

Metabolic-associated steatotic liver disease (MASLD) is the most prevalent liver disease worldwide,^1^^,^^2^^,^^3^ comprising a spectrum of histological conditions, including metabolic-associated steatotic liver (MASL) and its more progressive form metabolic-associated steatohepatitis (MASH). MASL is characterized by steatosis with or without inflammation and can progress to MASH when accompanied by lobular inflammation and hepatocyte ballooning.^4^ Patients with MASH develop fibrosis at twice the rate of patients with MASL and are at greater risk of progression to cirrhosis and hepatocellular carcinoma (HCC)^5^ and liver-related and overall mortality.^6^^,^^7^^,^^8^ MASLD diagnosis is typically incidental,^9^ and progression to more severe disease is typically asymptomatic, with patients often presenting later at end-stage liver disease with limited treatment options.^10^

Obesity is a major risk factor for MASLD, with ∼75% MASLD prevalence in individuals with obesity^11^ compared with 32.4% of the general population.^11^ Despite the high prevalence of MASLD, there remains a lack of practical, effective, and non-invasive screening options to identify early-stage disease before progression to cirrhosis, particularly in obese patients.^12^ The current gold standard for MASLD and MASH diagnosis is liver biopsy with histopathology.^13^ However, widescale use of liver biopsy is not practical given the time, risks of adverse events, and high volume of patients at risk of MASLD.^1^^,^^2^^,^^3^ There have been significant advancements in imaging modalities to diagnose MASLD, MASH, and advanced fibrosis. Magnetic resonance imaging (MRI) has strong predictability to diagnose hepatic fibrosis^14^ and has a modest ability to differentiate MASL from MASH.^15^^,^^16^ Similarly, transient elastography (TE) effectively stratifies obese patients with severe fat accumulation but is not effective at differentiating MASH from MASL.^17^ Both MRI and TE require visitation to specialized centers that is not practical for population-level screening.

To overcome these limitations, simple blood biomarkers and panels were developed to best identify and exclude significant to advanced liver fibrosis; these include Fibrosis-4 Index (FIB-4), Forns index, and commercial biomarker panels such as the enhanced liver fibrosis (ELF) test.^18^^,^^19^ However, these tests perform poorly in obese individuals^12^ and do not differentiate MASLD from MASH,^20^^,^^21^^,^^22^ and the biomarkers used in these panels are not reflective of hepatic MASH pathology,^12^ which is a pre-requisite for a reproducible and effective biomarker. Therefore, identifying liver-secreted proteins that reflect hepatic pathology and can effectively stratify patients at risk of MASH is of utmost importance.

The development of effective biomarkers and predictive panels should include consistent biological plausibility that reflects the underlying liver pathology occurring in MASH. Given that ∼25% of circulating proteins are predicted to be derived from the liver,^23^ and liver-secreted proteins are altered in murine models of MASLD,^24^^,^^25^ there is a high likelihood that a subset of liver-secreted proteins could be identified in human blood to effectively stratify patients with MASH. Previous findings reported correlations between liver gene expression and plasma proteomics^26^; however, liver gene expression correlates poorly with liver protein secretion in mice,^25^ questioning the utility of using gene expression to identify biomarkers of relevance to liver pathology in human MASH.

Mass spectrometry-based proteomics provides a comprehensive and unbiased assessment of proteins in a given tissue.^27^^,^^28^^,^^29^ Plasma is an easily accessible fluid that is effective for population-level screening.^30^ Plasma proteomics can facilitate biomarker discovery by simultaneously assessing thousands of candidate proteins that could effectively stratify patients with, or at risk of, a pathology.^26^^,^^29^^,^^31^^,^^32^^,^^33^ However, an inherent problem of plasma proteomics is mitigating the over 13 orders of magnitude of protein abundance^34^ that limits protein detection of low-abundant proteins, prompting the need for refined approaches.

In this context, we implemented independent high-sensitivity proteomic approaches to detect low- and high-abundant proteins with the aim of developing a biologically plausible non-invasive predictive model for MASH. Leveraging precision-cut technology, we performed a detailed assessment of human liver-secreted proteins using data-independent acquisition mass spectrometry in 96 individuals. We detected 3,333 secreted proteins from the human liver, of which 102 proteins were differentially secreted in individuals with MASH compared to individuals with no pathology. Incorporating the liver-secreted proteome and dual plasma proteomic approaches, we identified biomarkers for MASH in persons with obesity. We used these biomarkers to develop and validate a blood-based diagnostic multivariable index that can effectively stratify MASH or those without MASH in persons with obesity.

## Results

### Discovery cohort: Patient information

We recruited 160 bariatric patients (BMI 45.2 ± 7.7) and observed an incidence rate of 26.3% for no pathology, 53.1% for MASL, and 20.6% for MASH (Tables 1 and S1). There was an overall female predominance in our study (73.1%) but a higher rate of males with MASH than those with no pathology (42.4% vs. 16.6%). The incidence of type 2 diabetes mellitus (T2D) and hypertension was not different between groups. Alanine aminotransferase (ALT) and aspartate aminotransferase (AST) were increased with MASH compared to MASL but were not different compared to those with no pathology. High-density lipoprotein (HDL) was reduced in patients with MASH and MASL compared with no pathology. The levels of HbA1c and bilirubin were increased in MASH compared to patients with no pathology and MASL (Tables 1 and S1). Recent work indicates the existence of two distinct types of clinically relevant MASLD with similar liver phenotypes at baseline.^35^ Using the equation developed in this previous study, individuals with MASH were more likely to be clustered into a liver-specific adverse event compared to MASL (Table S1) and less likely to be clustered as a control compared to MASL and persons with no pathology (Table S1).

**Table 1:** Characteristics of the discovery cohort and comparisons between histologically defined groups

Parameter	Total (*n*)	No pathology (*n* = 42)	MASL (*n* = 85)	MASH (*n* = 33)	*p* value
Age (years)	160	45.9 ± 14.0	43.6 ± 10.0	40.9 ± 10.4	0.17
Gender (male, %)	160	7 (16.6%)	22 (25.9%)	14 (42.4%)	0.04
T2D (*n*, %)	160	6 (14.3%)	26 (30.6%)	8 (24.2%)	0.14
Hypertension (*n*, %)	160	15 (35.7%)	28 (32.9%)	12 (36.4%)	0.91
Weight (kg)	160	119.5 ± 21.9	128.4 ± 25.3	137.2 ± 33.2^a^	0.01
Pre-op weight loss (kg)	129	−4.8 ± 8.4	−5.9 ± 5.2	−5.3 ± 6.2	0.21
Pre-op weight loss (%/BW)	129	−3.7 ± 6.5	−4.2 ± 3.6	−3.5 ± 4.4	0.28
BMI (kg/m^2^)	159	43.4 ± 6.5	45.5 ± 7.6	46.7 ± 9.3	0.17
Glucose (mmol/L)	153	5.2 ± 1.0	5.7 ± 2.00	5.8 ± 2.2	0.22
ALT (U/L)	158	42.7 ± 60.4	39.4 ± 23.9	60.5 ± 37.9^b^	0.03
AST (U/L)	157	30.8 ± 25.8	30.4 ± 16.1	42.4 ± 28.0^b^	0.02
Triglycerides (mmol/L)	156	1.39 ± 0.59	1.56 ± 0.66	1.63 ± 0.76	0.25
HbA1c (%)	147	5.51 ± 0.42	5.85 ± 0.93	6.52 ± 1.75^a^^,^^b^	0.0007
HOMA2IR	139	1.32 ± 0.98	1.65 ± 1.47	1.61 ± 1.40	0.44
Histology	160	–	–	–	–
Steatosis score	160	–	–	–	–
0	–	42 (100%)	0 (0%)	0 (0%)	<0.001
1	–	0 (0%)	55 (64.7%)	5 (15.1%)	–
2	–	0 (0%)	29 (34.1%)	22 (66.7%)	–
3	–	0 (0%)	1 (1.2%)	6 (18.2%)	–
Inflammation score	160	–	–	–	–
0	–	39 (92.9%)	50 (58.8%)	0 (0%)	<0.001
1	–	3 (7.1%)	30 (35.3%)	29 (87.9%)	–
2	–	0 (0%)	4 (4.7%)	3 (9.1%)	–
3	–	0 (0%)	1 (1.2%)	1 (3.0%)	–
Ballooning score	160	–	–	–	<0.001
0	–	42 (100%)	78 (91.8%)	(0%)	–
1	–	0 (0%)	4 (4.7%)	6 (18.2%)	–
2	–	0 (0%)	3 (3.5%)	27 (81.8%)	–
NAS score	160	–	–	–	<0.001
≤2	–	42 (100%)	64 (75.3%)	0 (0%)	–
3–4	–	0 (0%)	21 (24.7%)	20 (60.6%)	–
≥5	–	0 (0%)	0 (0%)	13 (39.4%)	–
Fibrosis	160	–	–	–	<0.001
F0	–	42 (100%)	53 (62.4%)	15 (45.5%)	–
F1	–	0 (0%)	18 (21.2%)	15 (45.4%)	–
F2	–	0 (0%)	10 (11.8%)	2 (6.1%)	–
F3	–	0 (0%)	3 (3.5%)	1 (3.0%)	–
F4	–	0 (0%)	1 (1.2%)	0 (0%)	–

ALT, alanine aminotransferase; AST, aspartate aminotransferase; BMI, body mass index; BW, body weight; HOMAIR, homeostatic model assessment for insulin resistance; Pre-op, pre-operative; NAS, NAFLD activity score; T2D, type 2 diabetes mellitus.

a MASH vs. no pathology.

b MASH vs. MASL*.* Significance was determined by one-way ANOVA with Bonferroni’s multiple comparisons, chi-squared test, or pairwise Fisher’s exact test with Bonferroni’s multiple comparisons; see also Table S1. Data were expressed as mean ± standard deviation, and categorical variables were depicted as numbers with percentages within groupings.

### Effectiveness of current non-invasive scores to stratify MASH

We first assessed the areas under receiver operating characteristic (AUROCs) of non-invasive scores and clinical parameters that are commonly used to predict MASH compared to those without MASH (i.e., no pathology and MASL). Diagnostic accuracy for the Forns index, FIB-4 score, and plasma bilirubin all failed to stratify MASH from patients without MASH (Figures S1A–S1C). The AUROC of AST/ALT ratio (95% confidence interval [CI] 0.52–0.73, *p* = 0.024) and HbA1c (95% CI 0.50–0.74, *p* = 0.039) exhibited a poor ability to discern MASH (Figures S1D and S1E). We next investigated circulating levels of soluble triggering receptor expressed on myeloid cells 2 (TREM2) that was reported as a useful biomarker for MASH.^26^^,^^36^^,^^37^ The average concentration of TREM2 was 45.96 ng/mL across all patients. Individuals with MASL or MASH had increased plasma TREM2 compared with no pathology (Figure S1), but TREM2 was unable to stratify for MASH owing to its inability to discriminate MASH from MASL (Figures S1G–S1I). These data indicate limited utility of currently used non-invasive scores/clinical parameters and biomarkers for MASH prediction in obese persons.

### Dual plasma proteomic approaches identify candidate biomarkers for biopsy-proven MASH

**Figure 1:**
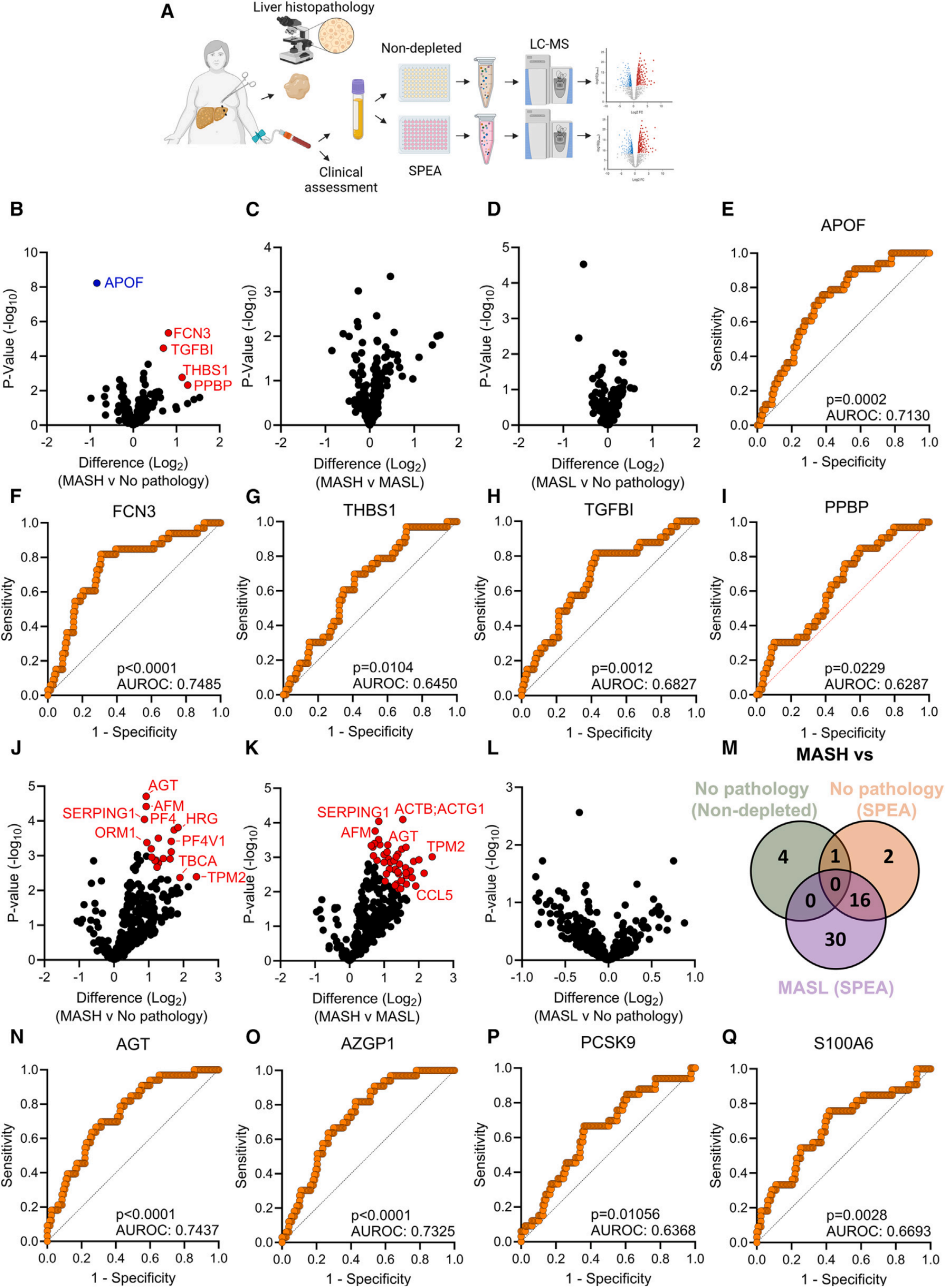
Dual proteomic approaches identify candidate biomarkers for MASH in the plasma proteome (A) Schematic of the biomarker discovery workflow. (B and C) Volcano plot of the non-depleted plasma proteome in patients with MASH (*n* = 33) when compared to those with (B) no pathology (*n* = 42) (C) and MASL (*n* = 85). (D–I) (D) Proteomic changes in MASL compared with no pathology. AUROC curves of candidate biomarkers to detect MASH: (E) APOF, (F) FCN3, (G) THBS1, (H) TGFBI, and (I) PPBP derived from the non-depleted proteomics. (J–L) Volcano plot of the SPEA plasma proteome in patients with MASH when compared to those with (J) no pathology and (K) MASL. (L) Volcano plot of the SPEA plasma proteomoe in patients with MASL when compared with no pathology. (M) Venn diagram of proteins significantly remodeled with MASH in the SPEA and non-depleted plasma proteome comparisons. (N–Q) AUROC curves to stratify MASH using the SPEA detected candidate biomarkers: (N) AGT, (O) AZGP1, (P) PCSK9, and (Q) S100A6. Data were determined by two-way t tests with Benjamini-Hochberg false discovery rate (adjusted *p* value < 0.05) or area under the receiver operative curve.

The patient recruitment and plasma proteomic workflow is described in Figure 1A. Plasma proteomic analysis using non-depleted plasma proteomics identified 234 high-abundant proteins. There was minor remodeling of the proteome when comparing MASH with no pathology (Figure 1B; Table S2), with an increase in ficolin 3 (FCN3), pro-platelet basic protein (PPBP), transforming growth factor β induced protein (TGFBI), and thrombospondin 1 (THBS1) and a reduction in apolipoprotein F (APOF). No differences were observed in the high-abundant plasma proteome when comparing MASH to MASL and MASL to no pathology (Figures 1C and 1D). After adjusting for age, gender, and BMI, partial correlation analysis identified 54 plasma proteins that were associated with histological components of MASH (Tables S3–S5). Sixteen plasma proteins were associated with steatosis, 23 with hepatocyte ballooning, and 15 with non-alcoholic fatty liver disease (NAFLD) activity score (NAS) (Figure S1F). No proteins were associated with lobular inflammation. APOF was the number one plasma protein correlated with steatosis, ballooning, and NAS (Figure S1J). The predictive capacity of each protein that was remodeled with MASH (Figure 1B) was assessed, and all potential biomarkers could stratify MASH at an acceptable (Figures 1E and 1F) or poor level (Figures 1G–1I). The predictive capacity for MASH stratification was validated for APOF and TGFBI, but not FCN3, PPBP, and THBS1, in another cohort of obese individuals with a NAS of >4 (Figure S1K–S1O).^26^

We next performed a small protein enrichment assay (SPEA) in the same patients to identify small, lower-abundant proteins that are infrequently detected with other plasma proteomics approaches.^38^ We detected 318 proteins, of which, 19 and 46 proteins were increased in MASH compared with no pathology (Figure 1J; Table S6) or MASL (Figure 1K), respectively. There were no differences in the plasma proteome when comparing MASL to those with no pathology (Figure 1L). Partial correlation analysis identified 95 proteins that were correlated with histological components of MASH (Tables S7, S8, and S9). Afamin (AFM), angiotensinogen (AGT), β-defensin 1 (DEFB1), and PRAP1 were correlated with steatosis; DEFB1 was correlated with the NAS; and 90 proteins were associated with hepatocyte ballooning (Figure S1P). No proteins were correlated with lobular inflammation. Sixteen plasma proteins were increased in MASH when compared to both MASL and no pathology (Figure 1M; Table S10). We termed these “MASH-regulated” plasma proteins. The most significantly upregulated proteins could stratify MASH with an AUROC ranging from 0.63 to 0.74 (Figures 1N–1Q), which outperformed other current non-invasive tests (Figure S1). These candidate biomarkers include AGT, zinc-alpha-2-glycoprotein (AZGP1), proprotein convertase subtilisin/kexin type 9 (PCSK9), and S100 calcium-binding protein A6 (S100A6). AGT, AZGP1, PCSK9, and S100A6 could stratify patients with a NAS of >4 in an independent cohort of obese persons (Figures S1Q–S1T).^26^

### The liver-secreted proteome is remodeled in MASH

**Figure 2:**
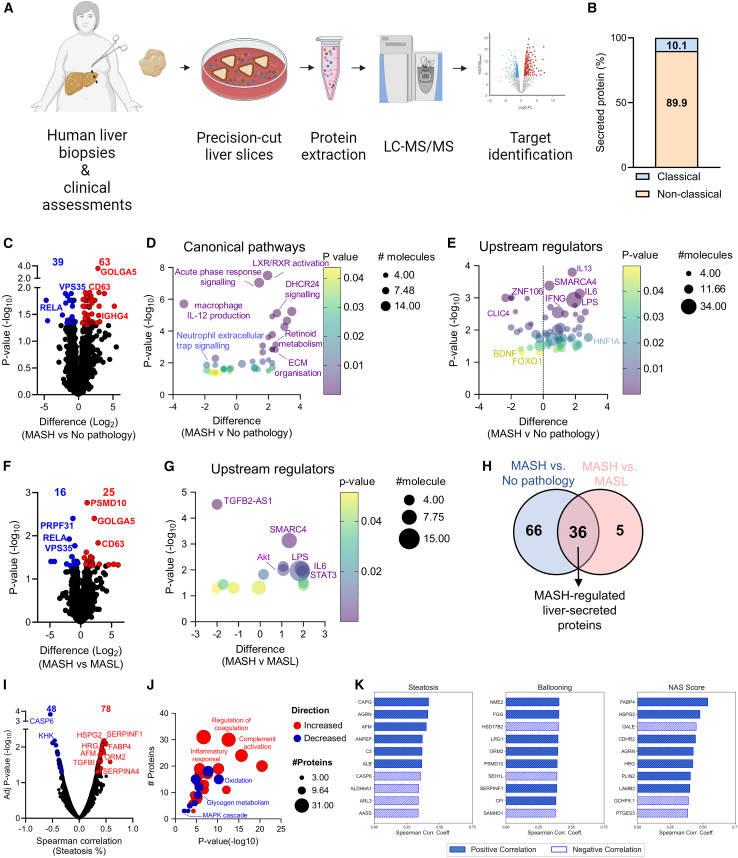
Protein secretion from the liver is remodeled in MASH (A) Schematic of the study design. (B) The percentage of classically and non-classically secreted proteins secreted from the human liver. (C–E) (C) Volcano plot of human liver-secreted proteins showing remodeling with MASH (*n* = 11) compared to no pathology (*n* = 26) and the ingenuity pathway analysis of (D) canonical pathways and (E) upstream regulators. (F and G) (F) Volcano plot depicting the proteins remodeled with MASH compared to MASL (*n* = 59) and (G) the ingenuity pathway analysis of upstream regulators. (H) Overlay of the liver-secreted proteins remodeled in livers with MASH. (I and J) (I) Spearman’s correlation analysis between the liver-secreted proteins and the pathologist-defined liver steatosis area and (J) the Metascape pathway enrichment analysis of steatosis correlated proteins. (K) Top 10 proteins that correlate with the Kleiner steatosis, ballooning, and nonalcoholic fatty liver disease (NAFLD) activity (NAS) scores, respectively. Significance was tested by two-way t tests or Spearman’s correlation with Benjamini-Hochberg false discovery rate (adjusted *p* value < 0.05).

Effective biomarkers should ideally exhibit biological plausibility that reflects changes in liver metabolism and viability and have predictable explanations to account for how interventions may alter their levels. To identify whether the candidate biomarkers discovered in the plasma proteomic screens were also liver secreted, we assessed protein secretion from precision-cut liver slices derived from a subset of patients in the discovery cohort (Table S11). In this experiment, a liver wedge was precision-cut to create 300 μm thick slices, which retain all liver cell types in their normal architecture (i.e., no tissue digestion) (Figure 2A). The slices were incubated for 16 h and the proteins secreted into the incubation medium were determined using liquid chromatography-tandem mass spectrometry (LC-MS/MS) with data-independent acquisition for broad coverage. We identified a total of 3,333 proteins in the secreted medium (Table S12), of which 10.1% were predicted to be classically secreted by the Human Protein Atlas (Figure 2B). There were no proteins exclusively detected in any one group. Sixty-three proteins were increased, and 39 proteins were decreased in the secreted medium with MASH compared with no pathology (Figure 2C). These proteins were associated with increases in liver X receptor (LXR)/ retinoid X receptor (RXR) activation that can regulate inflammation and lipid metabolism,^39^^,^^40^ extracellular matrix organization that is associated with the development of hepatic fibrosis,^41^ and DHCR24 signaling that can signal via LXR and protein kinase B (PKB/AKT)^42^ and, when inhibited, can ameliorate MASLD^40^ (Figure 2D). Bioinformatic prediction of upstream regulators identified an increase in interleukin (IL)-13 that can drive MASH-associated fibrosis^43^ and IL-6, which is associated with MASH progression^44^ (Figure 2E). When comparing MASH to MASL, the secretion of 25 proteins was increased and 16 were decreased (Figure 2F; Table S12). These proteins were predicted to upregulate mitogen-activated protein kinase cascade, which is involved in the transition from MASL to MASH^45^ and acute phase response signaling that is increased in MASH livers^46^ (Figure S2A). Analysis of upstream regulators predicted an increase in IL-6, which is frequently observed in MASH,^44^ and STAT3 that is inhibited by resmetirom,^47^ the only Food and Drug Administration-approved therapeutic for individuals with MASH^48^ (Figure 2G). We identified 36 liver-secreted proteins that were significantly altered when comparing the proteins that were significantly altered in both MASH vs. no pathology and MASH vs. MASL and termed these “MASH-regulated liver-secreted proteins” (Figure 2H; Table S13).

We next assessed the association between liver-secreted proteins and hepatic steatosis severity by performing pairwise global correlation analysis of liver-secreted proteins to the pathologist-defined steatosis area and histological features of MASH (Tables S14, S15, S16, and S17). We identified 78 secreted proteins that were positively associated with liver steatosis area (Figure 2I), with the top hits including fatty acid binding protein 4 (FABP4), kallistatin (SERPINA4), perlecan (HSPG2); all have known roles in lipid metabolism and driving MASLD progression.^49^^,^^50^^,^^51^ These positively associated proteins were predicted to be involved in the regulation of coagulation, inflammation, and complement activation (Figure 2J), features that are associated with MASH progression.^52^^,^^53^ Forty-eight proteins were negatively associated with steatosis area (Figure 2G) and were predicted to be involved in oxidation, which is often reduced in MASLD and MASH,^54^ and reduced glycogen metabolism that can increase lipid synthesis and MASLD^55^ (Figure 2J). To investigate correlations based on histological features of MASH, partial correlation analysis identified 69 proteins that were significantly correlated with stages of steatosis, 12 with ballooning and 10 with the NAS (Tables S15, S16, and S17). FABP4 and PLIN2 were identified among the top 10 proteins associated with NAS; these proteins were previously shown to correlate with alcoholic steatosis^56^ and can be predictive of the transition from MASL to MASH.^57^ The top 10 proteins correlated with each histological score are shown in Figure 2K. Together, these proteomic and correlational analyses enable a better understanding of hepatokine secretion associated with MASH, and the histological features of MASH in humans, as well as providing a basis for the discovery of candidate biomarkers for MASH.

### Identification of plasma biomarkers for MASH that reflect liver secretion

**Figure 3:**
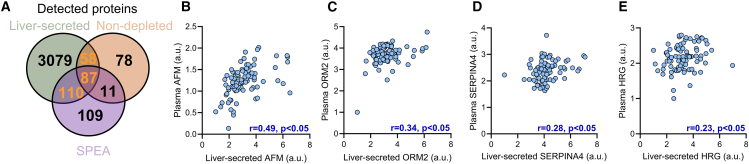
Identification of MASH biomarkers that reflect liver secretion (A) Overlay of proteins detected in the non-depleted and SPEA plasma proteome and the human liver-secreted proteome. Proteins detected in both liver-secreted and plasma proteomes are in orange numbers. (B–E) Correlation of the liver-secreted and plasma proteins detected using non-depleted proteomics: (B) AFM, (C) ORM2 (*n* = 85), (D) SERPINA4, and (E) HRG. Significance was tested by Pearson correlation. *n* = 86 unless stated otherwise.

To identify plasma biomarkers that reflect the underlying remodeling of MASH-induced liver secretion, we overlaid the three proteomic approaches and identified 255 proteins that were detected in both the liver-secreted proteome and one or both plasma proteomes (Figure 3A). Four proteins exhibited a significant increase in both liver secretion and plasma levels with MASH; these were AFM,histidine-rich glycoprotein (HRG), ORM2, and SERPINA4. Liver-secreted AFM, ORM2, and SERPINA4 correlated with their plasma levels using both proteomic approaches (Figures 3B–3E and S2B–S2D). Liver-secreted HRG levels weakly correlated with the plasma levels in the SPEA approach and were not associated with plasma levels when using the non-depleted proteome (Figures 3E and S2E).

### Integrative plasma proteomics effectively stratifies MASH in two bariatric cohorts

**Figure 4:**
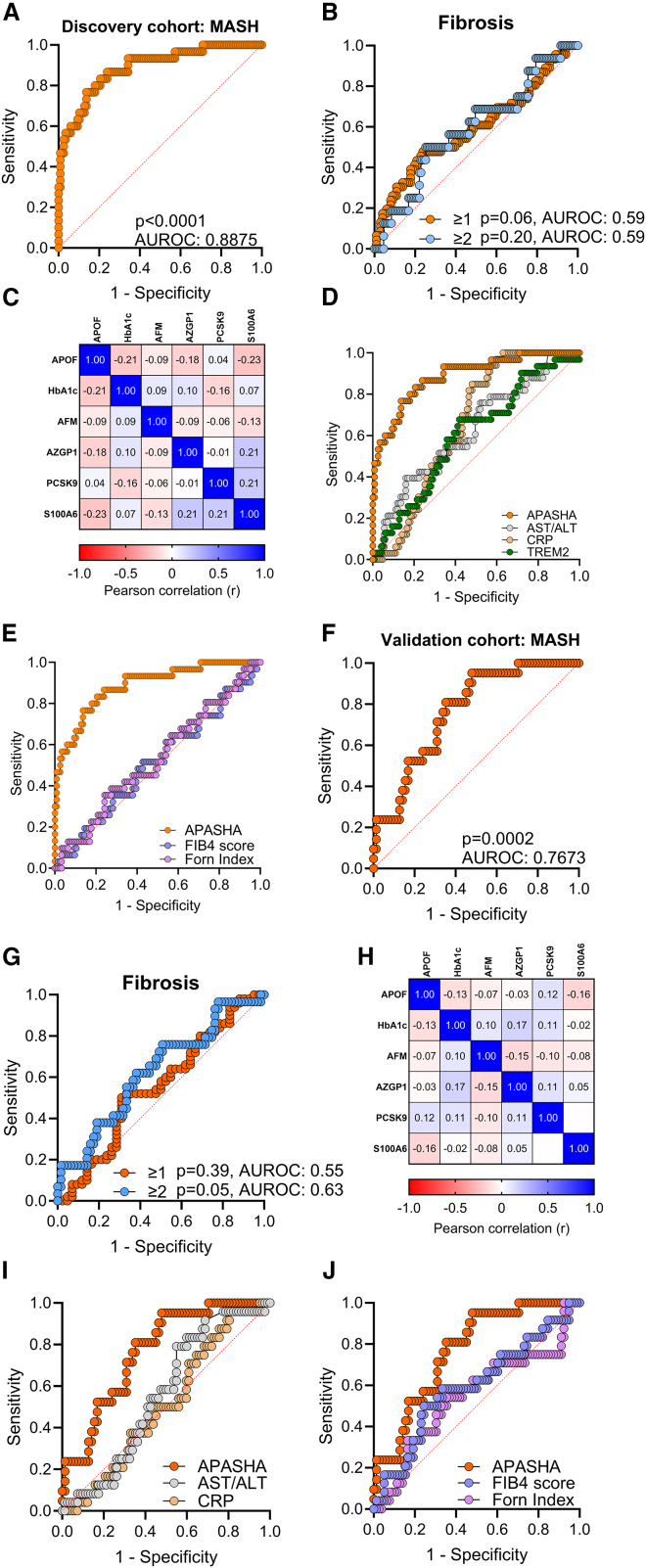
Diagnostic utility of the APASHA model to stratify MASH (A and B) (A) AUROC curve of the APASHA model in the discovery cohort to stratify individuals with MASH (*n* = 30) compared to No MASH (*n* = 117) and (B) hepatic fibrosis score ≥1 (*n* = 46) in orange or ≥2 in blue (*n* = 16). (C) Correlation matrix showing weak correlation between APASHA covariates in the discovery cohort. (D and E) (D) Diagnostic accuracy of the APASHA model vs. AST/ALT (MASH *n* = 31, No MASH *n* = 125), plasma TREM2 (MASH *n* = 31, No MASH *n* = 125), and CRP detected in the SPEA proteome (MASH *n* = 33, No MASH *n* = 127) and (E) non-invasive scores FIB-4 (MASH *n* = 31, No MASH *n* = 124) and Forns index (MASH *n* = 31, No MASH *n* = 124) to stratify for MASH in the discovery cohort. (F and G) (F) AUROC curve of the APASHA model in the validation cohort (MASH *n* = 21, No MASH *n* = 71) to stratify individuals with MASH and (G) hepatic fibrosis score ≥1 (*n* = 50) in orange or ≥2 (*n* = 29) in blue. (H) Correlation matrix showing weak correlation between APASHA covariates in the validation cohort. (I and J) (I) Diagnostic accuracy of the APASHA model vs. AST/ALT (MASH *n* = 24, No MASH *n* = 80) and CRP detected in the SPEA proteome (MASH *n* = 24, No MASH *n* = 82) and (J) non-invasive scores FIB-4 (MASH *n* = 24, No MASH *n* = 78) and Forns index (MASH *n* = 24, No MASH *n* = 79) in the validation cohort. Correlations were determined by Pearson correlation. Differences in AUROC were assessed by the DeLong test.

We next sought to develop an algorithm that could adequately stratify patients with MASH. Clinical parameters and plasma proteomics values were integrated, and a logistic backwards linear regression process was used to identify a biomarker-based model in the discovery cohort. The APASHA model—consisting of APOF, PCSK9, AFM, S100A6, HbA1c %, and AZGP1—showed an excellent discriminatory capacity with an AUROC of 0.8875 (CI 0.82–0.96) to discriminate MASH (Figure 4A; Tables S18 and S19), but not liver fibrosis (Figure 4B). Notably, inclusion of MASH-induced liver-secreted proteins ORM2, SERPINA4, and HRG (Figures 3C–3E) and gender did not improve model predictability and were eliminated through backward linear regression. All components of the APASHA model weakly correlated with each other indicating minimal interdependence (Figure 4C), and single-cell transcriptomics studies show that all proteomic biomarkers, except S100A6, are enriched in hepatocytes (Figure S3A).^58^ To facilitate clinical use, we established a threshold of >−1.217 to rule in MASH with 80% sensitivity, 82.05% specificity, a negative predictive value of 94.12%, and a likelihood ratio of 4.457 (Table S19). The APASHA model outperformed other non-invasive predictors including the AST/ALT ratio, plasma TREM2 levels and C-reactive protein (CRP, detected in the SPEA proteome) (Figure 4D), and the FIB-4 score and Forns Index (Figure 4E; Table S20).

To determine the external validity of our model, we prospectively recruited a validation cohort of persons undergoing bariatric surgery to assess the APASHA model’s discriminatory capacity. The validation cohort comprised 106 recruited persons, with an observed prevalence of 28.3% for no pathology, 49.1% for MASL, and 22.6% for MASH (Table S21). There was an overall female predominance (82%) in the validation cohort. The prevalence of hypertension was increased in persons with MASH. There were no differences in MASLD cardiometabolic and liver-specific endotypes between groups.^35^ Plasma triglycerides were elevated in persons with MASH compared to individuals with MASL and no pathology. ALT was increased in MASH and MASL individuals compared to those with no pathology.

The discriminatory capacity of the APASHA model was assessed in the validation cohort. The cohort consisted of 106 individuals with 92 HbA1c values (missing 11 values for no pathology and 3 for MASH). The APASHA model achieved an AUROC of 0.77 (CI 0.66–0.87) to discriminate MASH (Figure 4F; Table S19), but not liver fibrosis (Figure 4G). The individual components weakly correlated with each other (Figure 4H). In the independent cohort, APASHA performed better than liver damage and inflammation markers, the AST/ALT ratio and CRP (Figure 4I), and the Forns index and FIB-4 score (*p* = 0.059) (Figure 4J; Table S20).

## Discussion

We set out to identify plasma proteins with diagnostic value for the detection of MASH in obesity. We developed a comprehensive proteomic resource, utilizing two complementary plasma proteomics approaches that assessed high- and low-abundant plasma proteins and overlaid this with a comprehensive assessment of human liver-secreted proteins from individuals with obesity. We show mild MASH-induced remodeling of the non-depleted high-abundant proteome and major remodeling of the SPEA low-abundant and liver-secreted proteomes. By leveraging patient-matched liver-secreted and plasma proteomes, we identified liver-secreted proteins that correlated with circulating levels to reflect biological plausibility in MASH. Targets were integrated with clinical parameters to develop and validate the APASHA model, a blood-based risk stratification tool that reliably identifies MASH among patients with obesity, without other causes of liver disease or steatosis.

We focused on MASH given it is the more severe form of MASLD and is associated with accelerated progression of hepatic fibrosis and development of HCC.^21^ Effective biomarkers and predictive panels should include consistent biological plausibility, robust diagnostic performance, and identifiable risks for misclassification in the high-risk target population. Each individual circulating biomarker has strong association to predict MASH pathology. APOF and AFM are *bona fide* hepatokines^59^ with sole expression in hepatocytes and are thereby strongly positioned to reflect changes in hepatocyte metabolism and viability. AZGP1 and PCSK9 are enriched in the liver with the highest expression in hepatocytes.^59^ Further, liver-secreted AFM and AZGP1 correlate with circulating levels, adequately reflecting changes in liver secretion in MASH (Figures 3B, S2B, S2F, and S2G) and for liver-secreted AFM and liver steatosis (Figure 2I). HbA1c levels are commonly used alone or in combination with other components to predict MASLD^60^ with impairments in systemic glucose handling commonplace in individuals with MASH.^61^ S100A6 is an established plasma protein expressed by many cell types and is regulated by p53 and nuclear factor κB, both of which are increased in MASLD pathogenesis.^62^^,^^63^ Liver-specific single-cell transcriptomic studies corroborate these findings, highlighting the enrichment of APOF, PCSK9, AFM, and AZGP1 in hepatocytes, while S100A6 is expressed in hepatocytes and liver-resident immune cells, cholangiocytes, endothelial cells, and others.^58^ The discriminating strength of APASHA to detect borderline and “at risk” MASH was independent of age, gender, BMI, and fibrosis (Figures 3C, 3G, Tables S9, and S10), and all proteomic markers independently stratified for MASH in an external cohort.^26^ Incorporation of these biologically plausible targets is a strength of the APASHA model for screening and risk stratification.

A major strength of the study is the comprehensive proteomic resources assessing the high- and low-abundant proteins in the blood and the liver-secreted proteins across the MASLD disease spectrum. We detected 453 unique plasma proteins using the non-depleted plasma and SPEA approaches (Figure 2E), of which 255 (56%) were also detected in the liver-secreted proteome. These values are comparable to previous reports in humans with alcoholic liver disease where 77% of the identified plasma proteins were also detected in liver tissue.^31^ Capturing the changes in human hepatokine secretion is paramount to bridge the gap between human and murine studies^64^ and provides a clinically relevant unbiased depiction of the changes in hepatic protein secretion with steatosis and MASH progression. Livers with MASL have a steatosis score of ≥1–3 denoting liver steatosis area of 5% to over 66% of the histological area,^65^ representing significant variability in lipid accumulation, which may explain the lack of changes in protein secretion between MASL and no pathology (Table S12). Our pairwise correlation and partial correlation analysis revealed that liver steatosis modifies the secretion of 126 proteins, independent of alterations in inflammation, hepatocyte ballooning, and fibrosis (Figure 2I; Tables S14 and S15). This finding opens avenues for identifying potential targets and regulators in MASLD progression and potentially aiding in the development of hepatokine-targeted therapies.^64^ For example, kallistatin (SERPINA4) was recently shown to drive MASH in mice,^49^ and our clinical data add translatability by showing that kallistatin is increasingly secreted from human livers with increasing hepatic steatosis. It was surprising that only four liver-secreted proteins were increased with MASH with respect to liver secretion and circulating levels. This may indicate prominent autocrine and/or paracrine regulation of liver-secreted proteins and a low proportion of proteins entering the systemic circulation.^24^^,^^64^^,^^66^^,^^67^^,^^68^^,^^69^^,^^70^ The expansive resource of matched liver-secreted and plasma proteomics provides information to further interrogate the potential autocrine, paracrine, and endocrine role of liver-secreted proteins and their potential for clinical gain.

In contrast to other studies,^33^^,^^71^^,^^72^ we observed no differences in the plasma proteome when comparing MASL with no pathology. In concordance, the liver-secreted proteome was also not different when comparing MASL with no pathology. Our data suggest that, rather than lipid accumulation *per se*, more severe inflammation and perhaps hepatocyte cell death during the progression of MASH are required to alter the processes controlling liver protein secretion, which would in turn impact the plasma proteome. In support of this concept, partial correlation analysis identified major remodeling in plasma proteins when correlating hepatocyte ballooning with the plasma proteome (Tables S4 and S8). There are several plausible possibilities to explain the discordance between our results and previous studies, including the high specificity and sensitivity of our approaches and perhaps differences in diet. For instance, Australian’s typically consume fifty times less high-fructose corn syrup than those in the US, which would accelerate dysglycemia and MASH progression.^73^^,^^74^

The MASH-specific APASHA model offers several distinct advantages over other non-invasive tests. Unlike other models that are tailored to detect more advanced stages of liver disease (i.e., MASH F3/4) such as NIS4® and ELF, the APASHA model is specifically designed for screening and stratifying MASH in people with obesity. The development of this model focused on individuals with obesity, a group at higher risk for MASH, thus providing a broader and earlier detection scope to enable early interventions. The APASHA model outcompetes the AST/ALT ratio, the liver inflammation marker CRP, the commonly used MASH biomarker TREM2,^26^^,^^36^^,^^37^ and FIB-4, which was previously shown to predict at-risk MASH.^75^ Previous findings that defined MASH by NAS of ≥4 show that TREM2 is a non-invasive predictor of MASH.^37^ We show that TREM2 is increased with MASL and MASH but is unable to discern MASH from MASL (Figures S1F–S1I). TREM2 is increased with steatosis (score of 3),^37^ which can be present with or without inflammation and or ballooning, which may explain its inconsistency in this study. The APASHA model, with a MASH rule-in cutoff of >−1.217, achieved a negative predictive value of 94.12% in the discovery cohort and 85.24% in the validation cohort (Table S10), demonstrating a high usability to exclude patients that do not have MASH. Another inherent strength is that the APASHA model can stratify MASH, independent of fibrosis. As evident in our cohort (Tables 1 and S12) and others,^76^ liver fibrosis is not always present in patients with MASH. Thus, screening for MASH using the APASHA model provides a rationale to perform MRI or liver biopsy for diagnosis of liver histopathology and inform on liver fibrosis severity. Thus, the APASHA model has the potential to be an outstanding first test to direct patients with a higher risk of having MASH, independent of aminotransferases and other liver diseases. Hence, the clinical applicability of the APASHA model may be suitable in a primary care setting.

In summary, we characterized the liver-secreted and plasma proteins in patients with obesity and MASLD. These clinically relevant resources provide the foundation to deeply interrogate intra- and inter-hepatic communication and to identify potential targets for the treatment of MASLD and its comorbidities, as we have done previously using murine proteomic analysis.^24^^,^^25^^,^^66^^,^^68^^,^^77^ Leveraging the plasma proteomes and clinical information, we developed and validated the blood-based multivariable APASHA model that can effectively stratify MASH independent of fibrosis. External validation of these targets in other cohorts and development into antibody-based approaches have the potential for the APASHA model to assist healthcare providers to identify patients at risk of MASH and who require additional care.

### Limitations of the study

The patient demographic was largely Caucasian from Australian private and public hospitals. Given the increased risk of MASH in certain populations (e.g., Hispanic and possibly Indigenous Australians),^78^^,^^79^ future research should investigate whether the APASHA model is applicable to more diverse cohorts. Participants were recruited from bariatric cohorts, and very low-calorie diets (VLCDs) are often prescribed for three weeks prior to surgery to reduce body weight and liver size. VLCD reduced weight by < 5% and was not different between groups. While weight loss of this magnitude is generally insufficient to impact histopathology,^80^ the effects on the liver secretome and plasma proteome are unknown. Next, the inclusion of HbA1c into the APASHA model could impact the test reliability. Given the high likelihood of patients presenting with both MASLD and T2D,^81^ the use of glucose-lowering agents could reduce only HbA1c without impacting MASH or other parameters and hinder its predictability. We were unable to validate the APASHA model in previously published cohorts because HbA1c values were not available.^26^ Future investigations are required to better understand the impact of glucose-lowering therapies on the APASHA model. Other considerations, which may limit the generalizability of the conclusions, include the absence of genotype-mediated effects^82^ and the low number of persons with MASH in our liver-secreted proteome. Finally, mass spectrometry proteomics approaches may have limited immediate translatability; however, we note that mass spectrometry-based assays are increasingly implemented in various disciplines in clinical diagnostic laboratories. Alternatively, this issue could be overcome by the development of high-throughput enzyme immunoassays of APOF, AFM, AZGP1, PCSK9, and S100A6 in clinical diagnostic units.

## Resource availability

### Lead contact

Further information and requests for resources and reagents should be directed to and will be fulfilled by the lead contact, Matthew J. Watt (matt.watt@unimelb.edu.au).

### Materials availability

All reagents generated in this study are available from the lead contact with a completed Materials Transfer Agreement.

### Data and code availability

• All proteomics data have been deposited in ProteomeXchange database and are publicly available as of the date of publication. Accession numbers are listed in the key resources table.
• All code has been deposited at GitHub and is publicly available as of the date of publication. Accession numbers are listed in the key resources table.
• Any additional information required to reanalyze the data reported in this paper is available from the lead contact upon request.

## Acknowledgments

We thank the patients for their contribution to the study, Sydney MS at 10.13039/501100001774The University of Sydney for supporting the mass spectrometry analysis, and Jeff Molendijk for technical assistance. Figures 1A and 2A were created with BioRender.com. This study was funded by the 10.13039/501100000925National Health and Medical Research Council of Australia (NMHRC, APP1162511). W.D.N. was supported by 10.13039/501100001782The University of Melbourne Research scholarship. P.M.M. was supported by a Canadian Institutes of Health Research (10.13039/501100000024CIHR) post-doctoral fellowship and a 10.13039/501100000038Natural Sciences and Engineering Research Council of Canada (NSERC) post-doctoral fellowship. M.K.M. was supported by a Career Development Fellowship from the 10.13039/501100000925NHMRC (ID: APP1143224). B.L.P. was supported by an Emerging Leader Grant from the 10.13039/501100000925NHMRC (ID: APP2009642). O.L. is supported by an 10.13039/100015539Australian Government Research Training Program (RTP) Scholarship. No external body had influence over any aspect of the study or decision to submit for publication.

## Author contributions

W.D.N., A. Ryan, T.M.S., A. Rucinski, G.J.O., W.A.B., W.K., P.R.B., M.K.M., B.L.P., M.L., and M.J.W. contributed to the study conception, design, and implementation. W.D.N., Y.J., J.B., M.P., P.M.M., S.N.K., G.J.O., W.A.B., B.L.P., M.L., and P.R.B. were involved in data acquisition. W.D.N., O.L., B.L.P., A. Ryan, A. Rucinski, and T.M.S. performed data analysis. W.D.N. and M.J.W. drafted the initial version of the manuscript. All authors critically revised the manuscript, and all authors approve the final version. W.D.N. and M.J.W. are the guarantors and accept all responsibility for the work and the conduct of the study, have access to the data, and controlled the decision to publish.

## Declaration of interests

W.A.B. reports financial support for a bariatric surgery registry from the Commonwealth of Australia, Apollo Endosurgery, Covidien, Johnson and Johnson, Gore, and Applied Medical. She has also received a speaker’s honorarium from Merck Sharp and Dohme and a speaker’s honorarium and fees for participation in a scientific advisory board from Novo Nordisk. The Bariatric Registry and the honorariums are outside of the submitted work. M.J.W. has received financial support from Gilead Sciences and CSL.

## STAR★Methods

### Key resources table

**Table:** 

REAGENT or RESOURCE	SOURCE	IDENTIFIER
**Biological samples**		

Human plasma from obese individuals with varying degrees of MASLD	This paper	N/A

**Critical commercial assays**		

Human TREM2 ELISA	Abcam, Cambridge, UK	AB224881

**Deposited data**		

Set 1: Project Name: MASH Non-depleted plasma proteome	This paper	ProteomeXchange Project accession: PXD052784
Set 2: MASH SPEA plasma proteome	This paper	ProteomeXchange: Project accession: PXD052798
Set 3: MASH human liver - secreted proteome	This paper	ProteomeXchange: Project accession: PXD052787
Python scripts	This paper	https://github.com/willdepower/MASH_human_liver_plasma_proteomics.
Python scripts	Niu et al. ^56^	github.com/llniu/ALD-study
GepLiver: scRNAseq	Li et al. ^58^	https://doi.org/10.6084/m9.figshare.c.6223739.v1
Normalyser v 1.3.4.	Willforss et al. ^83^	https://github.com/ComputationalProteomics/NormalyzerDE

**Software and algorithms**		

Ingenuity Pathway analysis	QIAGEN	836507
Metascape	https://metascape.org/gp/index.html	N/A
GraphPad Prism (version 10)	GraphPad, USA	https://www.graphpad.com/scientific-software/prism/www.graphpad.com/scientific-software/prism/
R Version 4.3.2	R Development Core Team, 2016	https://www.r-project.org/
Python (version 2.0.3.1)	Python Software Foundation	https://www.python.org
Spectronaut v14	Biognosys, Schlieren.	https://biognosys.com/software/spectronaut/
Perseus (Version 1.6.10.50)	Tyanova et al.^84^	http://www.perseus-framework.org

### Experimental model and study participant details

#### Study approval & patient recruitment

Participants provided written and verbal informed consent. The study protocol conforms to the ethical guidelines of the 1975 Declaration of Helsinki and was approved by the University of Melbourne Human Ethics Committee (ethics ID 1851533), The Avenue Hospital Human Research Ethics Committee (Ramsay Health; ethics ID WD00006, HREC reference number 249), the Alfred Hospital Human Research Ethics Committee (ethics ID GO00005), and Cabrini Hospital Human Research Ethics Committees (ethics ID 09-31-08-15).

Eligible patients with obesity scheduled for primary or secondary sleeve gastrectomy, gastric bypass or the insertion of a laparoscopic-adjustable band were prospectively enrolled. Patients in the discovery and validation cohort were recruited concurrently and assessed separately. A detailed medical history was taken, and metabolic comorbidities were noted including the presence of previously diagnosed hypertension and diabetes assessed by oral glucose tolerance testing. Exclusion criteria included: age <18 years, previous gender reassignment, other causes of chronic liver disease and/or hepatic steatosis including Wilson’s disease, α-1-antitrypsin deficiency, viral hepatitis, human immunodeficiency virus, primary biliary cholangitis, autoimmune hepatitis, genetic iron overload, hypo- or hyperthyroidism, celiac disease, as well as recent (within three months of screening visit) or concomitant use of agents known to cause hepatic steatosis including corticosteroids, amiodarone, methotrexate, tamoxifen, valproic acid and/or high dose oestrogens.^13^ Further exclusion criteria included potential for alcohol induced liver disease, which was assessed through a modified version of the alcohol use disorders identification test (AUDIT).^13^^,^^84^ Ancestry, race, ethnicity, socioeconomic status data of participants were not collected.

### Method details

All patients were fasted for 8–12 h overnight and venous blood was taken before anesthesia. Patients were weighed on the day of their surgery and pre-operative weight loss was calculated from the weight of their initial consultation with their surgeon. Blood was transferred to 2 x K_2_E Ethylenediaminetetraacetic acid (EDTA), 2 x SST II Advance and 1 x FX 5 mg bio-containers for subsequent storage or clinical/biochemical assessments. All blood samples were sent to Melbourne Pathology (Victoria, Australia) for standardized measurement of biochemical and metabolic variables, except for one bio-container of EDTA. Standard blood analyses were performed for electrolytes, full blood examination, glucose, glycosylated hemoglobin (HbA1c), insulin, C-peptide, cholesterol, triglycerides, and liver function assessed by alanine aminotransferase (ALT), aspartate aminotransferase (AST), gamma glutamyltransferase (GGT) and alkaline phosphatase (ALP), and screening blood tests for liver disease. The FIB-4 Score and the Forn index were calculated as described in Table S13. The remaining blood within the EDTA tube was spun at 8000 x g for 10 min and the plasma was collected and stored at −80°C for mass spectrometry analyses.

#### Plasma proteomic studies

Human plasma samples from the discovery and validation cohorts were thawed on ice, mixed and an aliquot of 20 μL and 50 μL was transferred to 96 well plates (Eppendorf) for the high abundant non-depleted plasma proteomics approach^85^ and small protein enrichment assay proteome (SPEA) analysis, respectively. Plates were stored and shipped at −80°C prior to analysis.

#### Non-depleted plasma proteome digestion & analysis

Protein extraction, digestion, and clean-up of human plasma was performed as previously described.^85^ Briefly, one microliter of plasma was added to 24 μL of 1% sodium deoxycholate, 10 mM TCEP, 40 mM chloroacetamide, and 100 mM Tris-HCl (pH 8.5) in a 96-well plate (Eppendorf, Germany). Plates were sealed with a silicone mat (Eppendorf, Germany), and proteins were denatured, reduced, and alkylated by mixing on a Thermomixer-C (Eppendorf, Germany) with a ThermoTop (Eppendorf, Germany) at 95°C for 10 min at 1,000 rpm. Samples were cooled to room temperature and diluted to 10-fold with water. Proteins were digested into peptides with the addition of endoproteinase Lys-C and trypsin (both 1:100 ratio, μg/μg protein) and incubated at 37°C for 16 h at 1,000 rpm in an Eppendorf Thermomixer-C with a ThermoTop (heated lid). Peptides were diluted 2-fold with 99% ethyl acetate/1% TFA to form a 49.5% ethyl acetate/0.5% TFA solution.

SDB-RPS tips were washed with 1 x 100 μL of 100% ACN and centrifuged at 1000 x g for 1 min, and the flow-through was discarded. This was followed by addition of 1 x 100 μL of 0.1% TFA in water and 1 × 30% methanol/1% TFA in water, each wash step was followed by centrifugation at 1000 x g for 3 min and discarding of flow through. Next, tips were loaded with ∼10 μg of peptides and washed 2 x with 100 μL of 99% ethyl acetate/1% TFA and 1 x with 100 μL of 0.2% TFA in water. Peptides were eluted by addition of 100 μL of 5% ammonium hydroxide/80% ACN followed by centrifugation at 1000 x g for 5 min. Samples were dried using a GeneVac EZ-2 (Genevac, UK) at 40°C for 1 h. Dried peptides were resuspended in 30 μL of 5% (v/v) formic acid and stored at 4°C until analysis by LC-MS.

LC-MS was performed as previously described.^85^^,^^86^ An Exion LC system (Sciex, USA) was used to analyze peptide samples and was set up with a 5 cm × 2.1 mm, 1.9 μm particle, C18 column (Agilent, Australia), which was coupled to a Turbo-V ESI source on a Sciex 6600 mass spectrometer. The digested peptides (10 μg) in 5% (v/v) formic acid were directly injected onto the column and resolved over a gradient of 5–40% ACN at a flow rate of 1 mL/min for 5 min at 40°C. Peptides were ionised by electrospray ionization at 5.6 kV. The scanning SWATH acquisition was for 8 min per injection and scanned from 400 to 900 m/z with a 10 ms accumulation time.

Raw data were processed using DIA-NN 1.8^87^ with a search performed against the whole human proteome (Uniprot – canonical and isoform, downloaded 16/03/2021). A library-free strategy was employed with match between runs enabled. Cysteine carbamidomethylation was set as a fixed modification. N-terminal acetylation and methionine excision were set as variable modifications. Trypsin cleavage was allowed to have 1 missed cleavage event. Statistical analysis was performed on MaxLFQ normalized protein abundances^88^ grouped by MASH.

#### Plasma SPEA proteome approach

Plasma was processed using SPEA as previously described^38^ with some exceptions. Briefly, 50 μL of plasma was mixed by pipetting and incubated for 30 min at room temperature with 450 μL of ethanol-HCl buffer in the wells of an Agilent EMR Lipid 96-well SPE plate placed on top of a 500u μL protein lobing deepwell 96-well plate for sample collection. After incubation, the plate assembly was eluted by centrifugation according to manufacturer’s instructions. These eluates were used directly for the size exclusion separation of SPEA as described.^38^ After digestion and peptide cleanup as described,^38^ peptides were analyzed by LC-MS/MS on a NeoVanquish UHPLC coupled to an Exploris 480 mass spectrometer (Thermo Fisher Scientific). A 75 μm ID x 20 cm C18 pulled-tip column was used for peptide separation at 60°C, with sample loading at 850 bar (∼1.2 μL/min) and gradient separation from 3 to 50% B over 18 min at 300 nL/min. Mass spectrometry data acquisition used DIA at 15,000 resolution with 15 variable width windows spanning 423–892 m/z and the maximum injection time set to auto. Raw data were processed using Spectronaut (version 14) with a search performed against the whole human proteome. A library-free strategy was employed with match between runs enabled. Cysteine carbamidomethylation was set as a fixed modification. N-terminal acetylation and methionine excision were set as variable modifications. Trypsin cleavage was allowed to have 1 missed cleavage event. Statistical analysis was performed on normalized protein abundances grouped by MASH.

#### Candidate biomarker validation in external cohorts

The plasma Somascan data was taken from the supplementary material in Govaere et al. 2023.^26^ The plasma proteome was normalized using log_2_ transformation and mean normalization. Candidate biomarkers grouped based on a NAS score ≥4 (*n* = 79) as a surrogate marker for MASH compared to those without MASH (*n* < 4, *n* = 112). The diagnostic performance of each candidate biomarker to stratify for MASH was determined by the receiver operating characteristic (ROC) analyses and AUC calculations using GraphPad Prism (version 10.0).

#### Intraoperative liver biopsy & precision-cut liver slicing

An ∼1 cm^3^ wedge liver biopsy was collected from the left lobe of the liver during surgery. The liver was cut into two portions. One portion was placed in formalin and transported to TissuPath (Mount Waverley, Victoria), paraffin embedded and processed for histological analysis. Samples were graded according to the Clinical Research Network (CRN) NAFLD activity score (NAS)^65^ and Kleiner classification of liver fibrosis^4^ by a research active liver pathologist at TissuPath or Alfred Pathology. The main outcome was the diagnosis of NASH CRN where steatosis, inflammation, and ballooning scores are ≥1. The other portion of liver was placed in oxygenated Medium 199 media (M199; Gibco, USA) with 10% fetal bovine serum (FBS; Gibco, USA) and 1% penicillin-streptomycin (P/S; Gibco, USA), then embedded in 3% SeaPlaque agarose (Lonza BioScience, USA) using a Tissue Embedding Unit (Alabama Research and Development, USA). Embedded livers were then sliced on the Alabama R&D Tissue Slicer (Alabama Research and Development, USA) in 500 mL of oxygenated phenol red-free Dulbecco’s Modified Eagle Medium (DMEM; Gibco, USA) with 1% P/S at 300 μm thickness, as previously described.^24^^,^^66^^,^^67^ The liver slices were washed in Phosphate Buffered Saline (PBS; Gibco USA) and cultured in oxygenated M199 medium containing 1% P/S for 1 h. Subsequently, the liver slices were washed with PBS and cultured in 1 mL EX-CELL 325 protein free medium (Sigma-Aldrich, Australia) for 16 h at 37°C. The following day, the liver slices were weighed and snap-frozen in liquid nitrogen, while the incubation medium was collected and centrifuged at 300 x g at 4°C for 10 min. The supernatant was snap-frozen and stored at −80°C for subsequent proteomics analysis.

#### Liver-secreted proteomics

The assessment of liver-secreted proteins contained within the supernatant was performed as previously described.^24^ The supernatant was concentrated using Amicon Ultra-4 Centrifugal Filters (Merck, USA) at 4000 x g for 45 min, the concentrated sample washed in 2 mL 50 mM Tris-HCl, pH 8.0 + 150 mM NaCl at 4000 x g for 45 min, transferred to Eppendorf tubes and the protein content was determined by Pierce BCA protein assay kit (ID:23225, Thermofisher, USA). Protein disulphide bonds were reduced with the addition of 10 mM TCEP at 65°C for 20 min. The sample was mixed with 6.6x (v/v) of 5 M urea, added to Microcon-30kDa Centrifugal Filter (Sigma Aldrich, Australia), and centrifuged at 14,000 x g and 10°C for 15 min. This was followed by the addition of 200 μL of 5 M urea, centrifugation at 14,000 x g at room temperature for 15 min, and the flow-through was discarded. Samples were alkylated by the addition of chloroacetamide to a final concentration of 10 mM, and samples incubated for 20 min in the dark, and centrifuged at 14,000 x g. Proteins were washed 3 x with 100 μL of 5 M urea and 3x with 100 μL 50 mM ammonium bicarbonate (pH 8.5), and centrifuged at 14,000 x g and 10°C for 15 min and the flow through was discarded after each wash step. After the last wash step, a digestion solution was added to the centrifugal filter (1 μg Lys-C/100 μg protein in 75 μL of 50 mM ammonium bicarbonate for 2 h in a wet chamber (sealed plastic box with water rising 0.5cm from the bottom of the box) at 37°C. Subsequently, 2 μg trypsin/100 μg protein was added, and the samples were incubated in a wet chamber at 37°C for 16 h to allow for trypsin-mediated protein digestion. The following day, peptides were eluted from the centrifugal filters through the addition of 2 x 40 μL of 50 mM ammonia bicarbonate (pH 8.5) and 1 x 50 μL of 0.5 M NaCl; with each step being followed by centrifugation at 14,000 x g at room temperature for 15 min. The eluted peptides were acidified with 10% trifluoracetic acid (TFA) to a pH of 3, dried in a SpeedVac concentrator (Eppendorf Concentrator Plus, Germany) and stored at −80°C. SDB-RPS (polystyrene-divinylbenzene, reversed-phase sulfonate discs) (Sigma-Aldrich, Australia, Cat#66886- U) were doubled stacked and punctured with an 18-gauge needle and mounted in 200 μL tips (Eppendorf, Germany) to make SDB-RPS Stage tips. The filter tips were washed followed by the addition of 1 x 50 μL of acetonitrile (ACN), 1x 50 μL of 30% methanol, 1 x 50 μL 0.2% TFA and 1 × 1% TFA to the top of the tip and centrifugation at 1000 x g for 2 min, with the flow through discarded at each step. The dried-down peptides were resuspended in 50 μL of ACN, added to the top of the equilibrated filter tip and centrifuged at 1000 x g for 2 min. The peptides were washed once with 100 μL of 1% TFA and 99% ethyl acetate, and once with 100 μL of 5% ACN and 0.2% TFA in MilliQ-water. To elute the peptides 60 μL of 80% ACN/5% ammonium hydroxide (w:v) in water was added to each tip, the tips centrifuged at 1000 x g for 2 min and the eluted samples dried in a SpeedVac concentrator (Eppendorf Concentrator Plus, Germany). Dried peptides were resuspended in 30 μL of 2% ACN and 0.1% TFA and sent to the Charles Perkins Center (The University of Sydney, Australia) for liquid chromatography-tandem mass spectrometry (LC-MS/MS).

#### Mass spectrometry of liver secreted proteomics

Three microliters of peptides were injected and separated by Dionex 3500 ultra-high performance liquid chromatography (UHPLC, (Thermo fisher, USA)) coupled to a Q-Exactive HF-X mass spectrometer (ThermoFisher, USA) in the positive polarity mode as previously described.^89^ Peptides were resolved on a gradient set at 2–35% ACN containing 0.1% formic acid over 60 min at 800 nL/min, and separated on an in-house 100 μm × 20 cm column with an integrated emitter using a Sutter laser puller (1.9 μm particle size, C18AQ; Dr Maisch). Peptides were ionized by electrospray ionization at 2.3 kV. Data was acquired in positive ionization mode and the instrument was operated in data-independent acquisition mode (DIA). The DIA-MS method consisted of an MS1 scan that is acquired between 350 and 1650 m/z at a resolution of 30,000 and 3 x 10^6^ automatic gain control (AGC), with a 50 m injection time. Subsequently, using automatic injection and step normalized collision energies of 22.5, 25 and 27.5 at 15,000 resolution and 1 x 10^5^ AGC, 3 x 10^6^ AGC with higher-energy C-trap dissociation (HCD), 20 variable window sized DIA isolation and fragmentation occurred.^90^

#### Human liver-secreted proteomic data analysis

All DIA data was processed using Spectronaut v14 (Biognosys, Schlieren, Switzerland) with default settings employing retention time and mass recalibration. The default settings included retention time prediction type set to dynamic indexed retention time. Mass calibration was set to local mass calibration. Methionine oxidation and cysteine carbamidomethylation were set as variable modifications and fixed modifications, respectively. As previously described, interference correction on the MS1 and MS2 scans were enabled to remove fragments from quantification based on interfering signals, keeping at least three fragments per scan for quantification.^90^ The data was searched against the human Uniprot database (canonical and isoform – downloaded 16/03/2021) and the quantification was assessed on the MS2 extracted ion chromatograms. This was set at 3-6 fragment ions with >450 m/s. Labelling was set to label-free quantification with a minimum detection rate of 2. All other search parameters were default settings. The false discovery rate of the spectral peptide match to Uniprot proteins was set at 1%. The result output was further processed with Perseus (Version 1.6.10.50),^91^ a module from the MaxQuant suite.

#### Ingenuity Pathway analysis

Bioinformatic assessment of liver-secreted proteins grouped by MASH were performed using Ingenuity Pathway analysis (IPA, QIAGEN, Cat: 836507). IPA assessed canonical pathways and upstream regulators. The Gene ID, adjusted p-value and log_2_ fold change were inputted into the software. Data visualization was performed in Prism 10.0 (GraphPad, USA).

#### Metascape pathway analysis

Bioinformatic assessment of steatosis correlated liver-secreted proteins was performed using Metascape.^92^ Significantly correlated proteins defined as by adjusted P-value <0.05 were inputted into the software for pathway enrichment analysis. Data visualization was performed in Prism 10.0 (GraphPad, USA).

#### TREM2 ELISA

EDTA-plasma TREM2 levels were quantified using the Human TREM2 enzyme-linked immunosorbent assay kit (AB224881, Abcam, Cambridge, UK) as per the manufactures directions and as previously described.^37^ Briefly, 50 μL of the sample and standard were added to the plate, followed by 50 μL of the detector and capture antibody mixture that was incubated for 1 h at room temperature at 400 rpm. The samples were washed, 100 μL of 3,3′,5,5′-Tetramethylbenzidine solution was added, and the samples were placed in the dark for 10 min shaking at 400 rpm. The reaction was stopped by the addition of 100 μL of stop solution and the absorbance was measured at 450 nm using a spectrophotometer (CLARIOstar Nano, BMG LABTECH, Germany).

#### Cell type enrichment

To determine the cellular sources of the APASHA model protein constituents, we referenced the GepLiver database that has integrated human liver single cell transcriptomic data from 347 individuals.^58^

### Quantification and statistical analysis

In this study, statistical significance was defined as a *p*-value or adjusted *p*-value less than 0.05. N corresponds to the number of individuals under each grouping. For continuous variables, normality was assessed using Shapiro-Wilk test. Parametric data was expressed as mean ± standard deviation. Categorical variables were depicted as numbers with percentages within groupings. Pearson chi-squared test or Fisher’s exact test was used for independent categorical variables. Statistical analysis was performed using unpaired Student’s t test or one-way ANOVA for normally distributed data in R (version 4.3.2; pROC package, version 1.18.5), Python 3 (version 3.11) or GraphPad Prism. Means were compared using Bonferroni post hoc analysis for clinical biochemistry. Non-parametric data was expressed as mean ± SE and analyzed using the Kruskal-Wallis test with Dunn’s multiple comparisons. Statistical details can be found in the relevant figures, and accompanying figure legends and supplementary tables.

#### Plasma proteomic data analysis

The plasma proteomic data was uploaded into Perseus version 2.0.3.1,^91^ a module from the MaxQuant suite, and the data was normalized using Log_2_ transformation and mean normalization. Samples with evidence of significant hemolysis were excluded from all plasma proteomic analyses (*n* = 26). Plasma proteome datasets were filtered for 50% valid values across all samples with the remaining missing values imputed by drawing random samples from a normal distribution with downshifted mean by 1.8 s.d. and scaled s.d. (0.3) relative to that of abundance distribution of all proteins in one sample. Subsequently, students 2-way t-tests with Benjamini-Hochberg false discovery rate was performed and significant proteins were assigned based on q < 0.05.

#### Partial correlation analysis

Plasma and liver-secreted proteome correlation to histology scores was performed as previously described^56^ with minor modifications. For correlation analysis, imputation was performed on liver-secreted proteomic data as described in plasma proteomics in Python.^56^ No imputation was performed on any clinical variables. The python script that was previously developed to handle ANCOVA proteomics and multiple hypothesis testing^56^ using the statistical package pingouin (v 0.4.0) was applied to determine the spearman correlation coefficient controlled for the co-variates age, sex and BMI. Correlation was considered significant if the adjusted P-value, determined by FDR Benjamini-Hochberg was <0.05 and r≥0.3 or ≤-0.3. Pairwise correlation analysis of liver-secreted proteins to pathologist-defined steatosis percentage was performed in Python 3 using Pandas, NumPy, SciPy, and Statsmodels. Spearmans rank order correlation coefficient was calculated comparing the liver-secreted proteins with their steatosis percentage area using the spearmanr function in SciPy library and corrected for multiple comparisons using the Statsmodel library with the Benjamini-Hochberg procedure for false discovery rate (FDR) correction. All proteomic correlation data was performed in the Jupyter notebook environment. Data visualization was performed in Python 3 (version 3.11) and Graphpad Prism 10.0.

#### Liver-secreted proteome data analysis

Statistical analysis of liver-secreted proteomic data and differential expression analysis MASH, stratifications were Log_2_ transformed and median normalized. Differential expression analyses of proteins by MASH severity (e.g. MASH vs No pathology) was conducted using Normalyser software (version 1.3.4)^83^ and assessed with a multi-group comparison by using median normalization and robust linear regression and linear models for microarray with a one-way analysis of variance (ANOVA) and 2-way comparisons against each group using the program. This allowed the estimation of variance of each protein and reporting of the differential expression (or amounts) in secreted proteins between all groups. Subsequently, students 2-way t-tests with Benjamini-Hochberg false discovery rate was performed and significant proteins were assigned based on q<0.05. Statistical significance was defined as an adjusted p-value < 0.05. Data visualization was performed in Prism 10.0 (GraphPad, USA).

#### Outcome and predictor variables

The main outcome was the diagnosis of the NASH-CRN defined MASH, where steatosis, inflammation, and ballooning scores were all ≥ 1 giving a NAS ≥ 3.^4^ Patients who did not meet this criterion were grouped as No MASH. The modeling was developed on a dataset matrix comprising of 160 patients in the discovery cohort, using a total of 9 clinical and 32 proteomic continuous variables and 2 discrete variables (type 2 diabetes & sex). The variables were included if they were altered in either the clinical biochemistry, the plasma proteomic analyses (p<0.05) and/or were known *bona-fide* hepatocyte secreted proteins defined by the Human Protein Atlas^59^ that were significantly correlated with their plasma levels.

#### Development and training of the APASHA algorithm

The 43 predictor variables from the clinical parameters and the biomarker candidates were retained. A correlation matrix was performed to exclude covariates that were moderately correlated (Pearson’s coefficient ≥ 0.35 or ≤ -0.35). The remaining subset of proteomic and clinical variables were used for binary logistic regression eliminating the least predictive variable each time until the model identified patients with MASH (cut off *p* < 0.1) to generate the APASHA model:

APASHA model = −13 + (−0.8873 x APOF) + (0.5208 x HbA1c%) + (2.547 x AFM) + (1.239 x AZGP1) + (−1.085 x PCSK9) + (0.6684 x S100A6)

The overall diagnostic performance of non-invasive scores, panels and the APASHA model was determined by the receiver operating characteristic (ROC) analyses and AUC calculations using GraphPad Prism (version 10.0). Differences in AUROC were assessed using the DeLong test in R (version 4.3.2, pROC package, version 1.18.5) and graphed in GraphPad Prism. In the discovery cohort, nine patients with No MASH and 3 with MASH did not have HbA1c values and were excluded from the analysis. In the validation cohort, eleven patients with No MASH and 3 with MASH did not have HbA1c values and were excluded from the analysis.

## Supplemental information

Document S1. Figures S1–S3 and Tables S3–S5, S7, S9, S10, S13, S16–S20, and S22

Table S1. Full description of the discovery cohort and comparison between histologically defined groups, related to Figure 1 ALT, alanine aminotransferase; AST, aspartate aminotransferase; BMI, body mass index; HOMAIR, homeostatic model assessment for insulin resistance; NAS, NAFLD activity score; T2D, type 2 diabetes mellitus. Significance was determined by one-way ANOVA with Bonferroni’s multiple comparisons, chi-squared test, or pairwise Fisher’s exact test with Bonferroni’s multiple comparisons. MASLD endotypes were determined by Raverdy et al. (PMID: 39653777). Significance set at *p* < 0.05. ∗MASH vs. no pathology, †MASH vs. MASL. Data were expressed as mean ± standard deviation, and categorical variables were depicted as numbers with percentages within groupings.

Table S2. Non-depleted human plasma proteome, related to Figure 1

Table S6. The SPEA plasma proteome, related to Figure 1

Table S8. Proteins significantly correlated with ballooning grade from the SPEA plasma proteome, related to Figure 1

Table S11. Characteristics of the liver-secreted proteome cohort and comparison between histologically defined groups, related to Figure 2 ALP, alkaline phosphatase; ALT, alanine transferase; AST, aspartate transferase; BMI, body mass index; eGFR, estimated glomerular filtration rate; GGT, gamma-glutamyl transferase; Hb, hemoglobin; HbA1c, glycosylated hemoglobin; HDL, high-density lipoprotein; LDL, low-density lipoprotein; MCV, mean corpuscular volume; MCH, mean corpuscular hemoglobin; MCHC, mean corpuscular hemoglobin concentration; TSH, thyroid-stimulating hormone; WCC, white cell count. ∗ indicates significant difference (p < 0.05) compared to no pathology. ⸸ denotes significant difference compared to MASL as determined by one-way ANOVA with Bonferroni’s multiple comparisons or chi-squared test. Significance set at *p* < 0.05. Data were expressed as mean ± standard deviation, and categorical variables were depicted as numbers with percentages within groupings.

Table S12. The human liver-secreted proteome, related to Figure 2

Table S14. Proteins significantly correlated with the pathologist-defined steatosis percentage from the liver-secreted proteome, related to Figure 2

Table S15. Proteins significantly correlated with steatosis grade from the liver-secreted proteome, related to Figure 2

Table S21. Characteristics of the validation cohort and comparison between histologically defined groups, related to Figure 4 ALT, alanine aminotransferase; AST, aspartate aminotransferase; BMI, body mass index; HOMAIR, homeostatic model assessment for insulin resistance; NAS, NAFLD activity score; T2D, type 2 diabetes mellitus. Significance was assessed by one-way ANOVA with Bonferroni’s multiple comparisons or chi-squared test. MASLD endotypes were determined by Raverdy et al.^35^ (PMID: 39653777). Significance set at *p* < 0.05. MASH vs. no pathology, MASH vs. MASL. Data were expressed as mean ± standard deviation, and categorical variables were depicted as numbers with percentages within groupings.

Document S2. Article plus supplemental information
