# Supplementary material for: Integrated liver-secreted and plasma proteomics identify a predictive model that stratifies MASH
Source: Cell Rep Med. 2025 Apr 17;6(5):102085. doi: 10.1016/j.xcrm.2025.102085 (PMC12147855; doi:10.1016/j.xcrm.2025.102085)
Supplement: Document S1. Figures S1–S3 and Tables S3–S5, S7, S9, S10, S13, S16–S20, and S22 [file mmc1.pdf]

Cell Reports Medicine, Volume *6*

Supplemental information

Integrated liver-secreted and plasma proteomics identify a predictive model that stratifies MASH

William De Nardo, Olivia Lee, Yazmin Johari, Jacqueline Bayliss, Marcus Pensa, Paula M. Miotto, Stacey N. Keenan, Andrew Ryan, Amber Rucinski, Tessa M. Svinos, Geraldine J. Ooi, Wendy A. Brown, William Kemp, Stuart K. Roberts, Benjamin L. Parker, Magdalene K. Montgomery, Mark Larance, Paul R. Burton, and Matthew J. Watt

Supplementary information

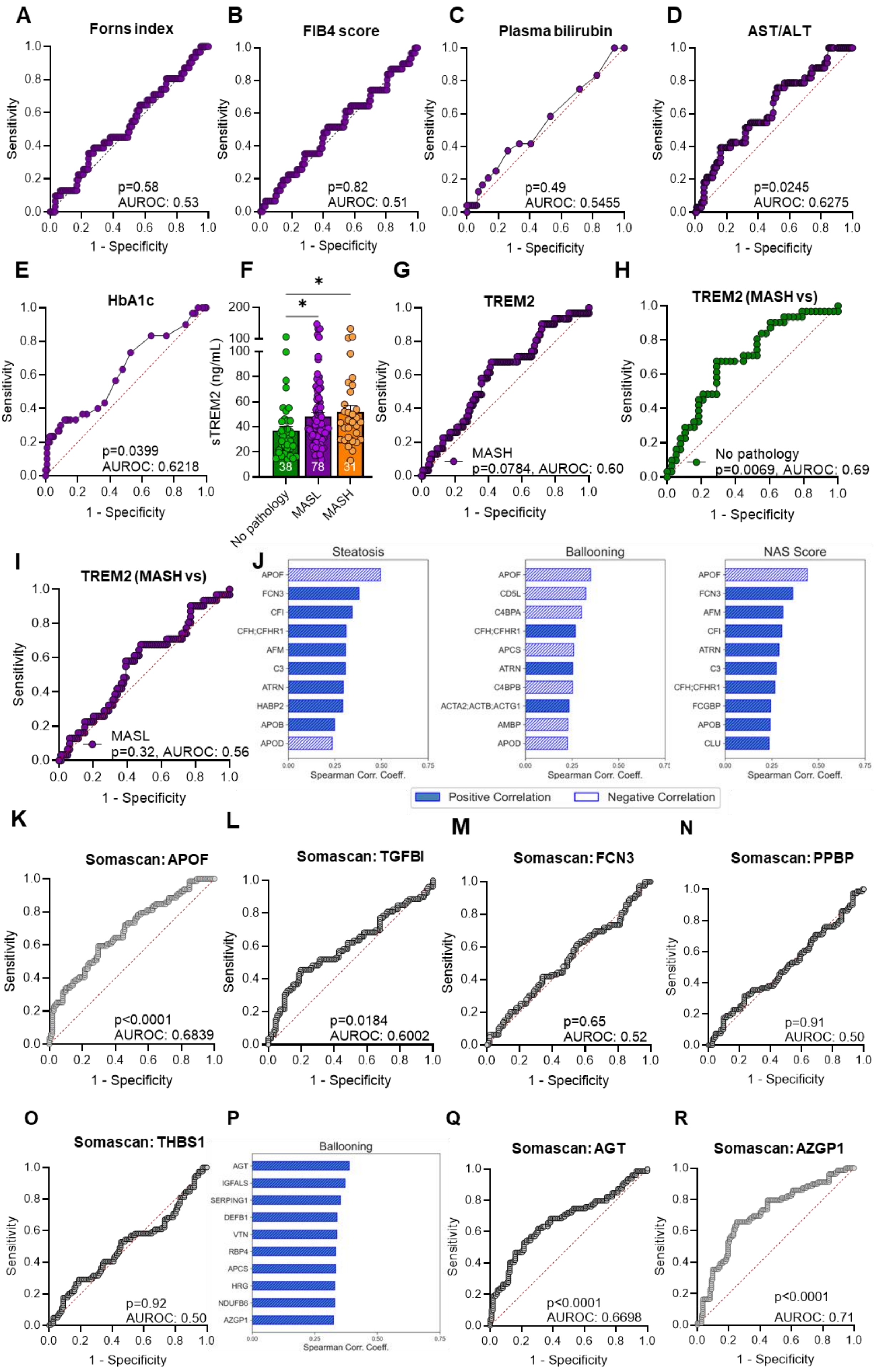
A
Forns index
p=0.58
AUROC: 0.53
B
FIB4 score
p=0.82
AUROC: 0.51
C
Plasma bilirubin
p=0.49
AUROC: 0.5455
D
AST/ALT
p=0.0245
AUROC: 0.6275
E
HbA1c
p=0.0399
AUROC: 0.6218
F
sTREM2 (ng/mL)
38
78
31
No pathology
MASL
MASH
G
TREM2
MASH
p=0.0784, AUROC: 0.60
H
TREM2 (MASH vs)
No pathology
p=0.0069, AUROC: 0.69
I
TREM2 (MASH vs)
MASL
p=0.32, AUROC: 0.56
J
Steatosis
APOF
FCN3
CFI
CFH;CFHR1
AFM
C3
ATRN
HABP2
APOB
APOD
Ballooning
APOF
CD5L
C4BPA
CFH;CFHR1
APCS
ATRN
C4BPB
ACTA2;ACTB;ACTG1
AMBP
APOD
NAS Score
APOF
FCN3
AFM
CFI
ATRN
C3
CFH;CFHR1
FCGBP
APOB
CLU
Spearman Corr. Coeff.
Positive Correlation
Negative Correlation
K
Somascan: APOF
p<0.0001
AUROC: 0.6839
L
Somascan: TGFBI
p=0.0184
AUROC: 0.6002
M
Somascan: FCN3
p=0.65
AUROC: 0.52
N
Somascan: PPBP
p=0.91
AUROC: 0.50
O
Somascan: THBS1
p=0.92
AUROC: 0.50
P
Ballooning
AGT
IGFALS
SERPING1
DEFB1
VTN
RBP4
APCS
HRG
NDUFB6
AZGP1
Spearman Corr. Coeff.
Q
Somascan: AGT
p<0.0001
AUROC: 0.6698
R
Somascan: AZGP1
p<0.0001
AUROC: 0.71
Sensitivity
1 - Specificity

S T

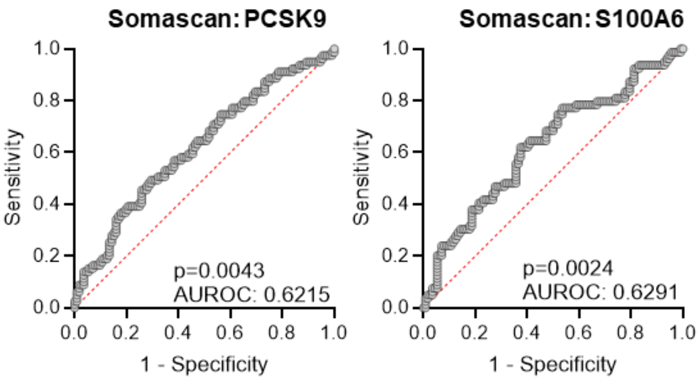

Figure S1. Current non-invasive scores and clinical parameters fail to stratify MASH, related to Figure 1.

(A) AUROC showing diagnostic accuracy to stratify MASH of current non-invasive scores of the forn index, **(B)** Fib4 score, **(C)** plasma bilirubin, **(D)** AST/ALT ratio, **(E)** HbA1c. **(F)** Plasma TREM2 levels stratified by MASH. **(G)** Diagnostic accuracy of plasma TREM2 to stratify MASH, and to stratify **(H)** MASH against only those with No pathology and **(I)** only those with MASL. **(J)** The top 10 non-depleted proteins correlate with Steatosis, Ballooning and NAS grade, respectively. **(K)** Independent plasma proteome AUROC curves showing diagnostic accuracy to stratify MASH (NAS≥4, n=79) compared to No MASH (n<4, n= 112)[1] of APOF, **(L)** TGFBI, **(M)** FCN3, **(N)** PPBP **(O)** THBS1. **(P)** The top 10 SPEA plasma proteins denoted by Gene ID that correlate hepatocyte ballooning grade, respectively. **(Q)** Independent plasma proteome AUROC curves showing diagnostic accuracy to stratify MASH (NAS≥4, n=79) compared to No MASH (n<4, n= 112) [1] of AGT, **(R)** AZGP1, **(S)** PCSK9 and **(T)** S100A6. Data are represented as mean ± SEM and analysed using Kruskal-Wallis test with Dunns multiple comparisons. N numbers depicted in bar graphs. Significance defined as $p<0.05$.

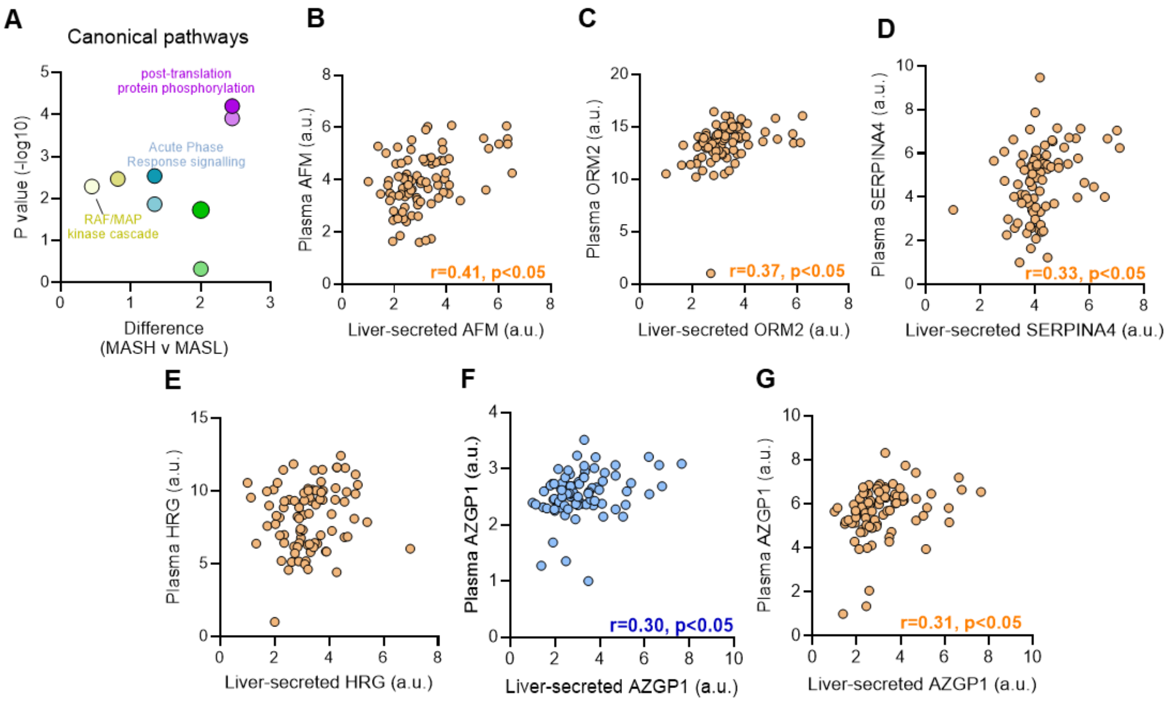

Figure S2. Correlation of liver-protein secretion and plasma proteome levels, related to Figure 3.

(A) Ingenuity Pathway analysis of canonical pathways altered with MASH compared to MASL. **(B)** Correlation of the liver-secreted and plasma proteins in patient-matched samples detected using proteomics for AFM (n=86), **(C)** ORM2 (n=85), **(D)** SERPINA4 (n=86), **(E)** HRG (n=86), **(F)** AZGP1 (n=85), and, **(G)** AZGP1 (n=85). Plasma proteins detected in the SPEA approach are depicted in orange and detected by non-depleted proteomics in blue. Significance tested by Pearson correlation.

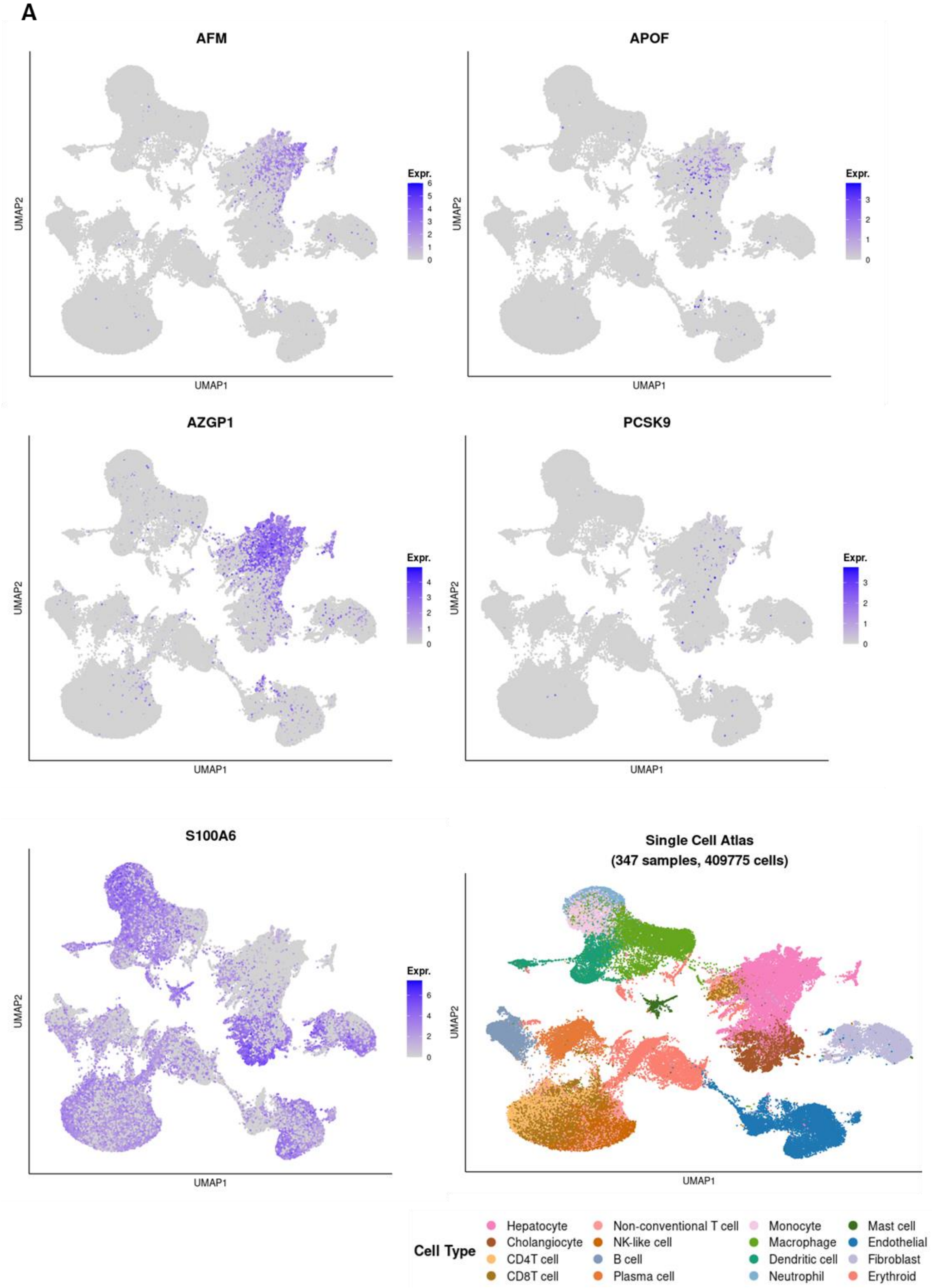

Figure S3. Identification of the cellular sources of APASHA protein constituents, related to Figure 4.

(A) Single cell RNA sequencing from the GepLiver single cell atlas identifying liver cell types that are enriched for APOF, PCSK9, AFM, AZGP1 and S100A6.[2]

Supplementary Table 3: Proteins significantly correlated to steatosis score from the non-depleted plasma proteome, related to Figure 1.

**n**	**r**	**CI95%**	**p-val**	**protein**	**pval_corrected**	**significant**
159	0.308353	[0.16 0.44]	8.99E-05	AFM	0.00313	TRUE
159	0.248513	[0.1 0.39]	0.001759	APOB	0.029398	TRUE
159	-0.23581	[-0.38 -0.08]	0.003042	APOD	0.044494	TRUE
159	-0.4952	[-0.61 -0.37]	4.97E-11	APOF	1.16E-08	TRUE
159	0.295602	[0.15 0.43]	0.000179	ATRN	0.004693	TRUE
159	0.307628	[0.16 0.44]	9.36E-05	C3	0.00313	TRUE
159	0.311358	[0.16 0.45]	7.61E-05	CFH;CFHR1	0.00313	TRUE
159	0.342426	[0.2 0.47]	1.21E-05	CFI	0.000945	TRUE
159	0.379969	[0.24 0.51]	9.96E-07	FCN3	0.000117	TRUE
159	0.292385	[0.14 0.43]	0.000212	HABP2	0.00497	TRUE
159	0.266506	[0.11 0.41]	0.000771	LGALS3BP	0.015655	TRUE
159	0.237639	[0.08 0.38]	0.002816	PROZ	0.043929	TRUE
159	0.265658	[0.11 0.41]	0.000803	SERPINA10	0.015655	TRUE
159	0.257515	[0.1 0.4]	0.001173	SERPINF1	0.021111	TRUE
159	0.295481	[0.15 0.43]	0.000181	TGFBI	0.004693	TRUE
159	0.32869	[0.18 0.46]	2.8E-05	VTN	0.001637	TRUE

Supplementary Table 4: Proteins significantly correlated to ballooning activity scores from the non-depleted plasma proteome, related to Figure 1.

n	r	CI95%	p-val	protein	pval_corrected	significant
159	0.23484	[0.08 0.38]	0.003168	ACTA2;ACTB;ACTG1	0.041185	TRUE
159	-0.2282	[-0.37 -0.07]	0.004167	AMBP	0.045379	TRUE
159	-0.25914	[-0.4 -0.11]	0.001088	APCS	0.021222	TRUE
159	-0.22548	[-0.37 -0.07]	0.004653	APOD	0.04734	TRUE
159	-0.34988	[-0.48 -0.2]	7.56E-06	APOF	0.000885	TRUE
159	0.254647	[0.1 0.4]	0.001337	ATRN	0.02353	TRUE
159	-0.30008	[-0.44 -0.15]	0.000141	C4BPA	0.005508	TRUE
159	-0.2535	[-0.39 -0.1]	0.001408	C4BPB	0.02353	TRUE
159	-0.32383	[-0.46 -0.18]	3.73E-05	CD5L	0.002181	TRUE
159	0.267187	[0.11 0.41]	0.000747	CFH;CFHR1	0.017472	TRUE
159	-0.23659	[-0.38 -0.08]	0.002944	CFP	0.041185	TRUE
159	-0.30561	[-0.44 -0.16]	0.000105	F2	0.004897	TRUE
159	0.381442	[0.24 0.51]	8.97E-07	FCN3	0.00021	TRUE
159	0.236006	[0.08 0.38]	0.003017	HABP2	0.041185	TRUE
159	-0.26508	[-0.41 -0.11]	0.000825	IGHG1	0.017549	TRUE
159	-0.34119	[-0.47 -0.19]	1.31E-05	IGHV1-3;IGHV1-46;IGHV1-69	0.00102	TRUE
159	-0.27527	[-0.41 -0.12]	0.000505	IGHV3-43D	0.013141	TRUE
159	-0.29197	[-0.43 -0.14]	0.000217	IGHV3-7;IGHV3-74	0.007256	TRUE
159	-0.2322	[-0.38 -0.08]	0.003536	IGLV3-25	0.043553	TRUE
159	-0.22798	[-0.37 -0.07]	0.004204	RBP4	0.045379	TRUE
159	0.227623	[0.07 0.37]	0.004266	SERPINF1	0.045379	TRUE

159	0.241301	[0.09 0.38]	0.002409	TGFBI	0.037576	TRUE
159	0.28739	[0.14 0.43]	0.000275	VTN	0.008047	TRUE

Supplementary Table 5: Proteins significantly correlated to NAS from the non-depleted plasma proteome, related to Figure 1.

n	r	CI95%	p-val	protein	pval_corrected	significant
159	0.3091	[0.16 0.44]	8.63E-05	AFM	0.005049	TRUE
159	0.242291	[0.09 0.38]	0.002308	APOB	0.03858	TRUE
159	-0.4406	[-0.56 -0.3]	8.61E-09	APOF	2.02E-06	TRUE
159	0.288162	[0.14 0.43]	0.000264	ATRN	0.008839	TRUE
159	0.274495	[0.12 0.41]	0.000525	C3	0.015353	TRUE
159	0.266828	[0.11 0.41]	0.00076	CFH;CFHR1	0.019748	TRUE
159	0.303949	[0.15 0.44]	0.000115	CFI	0.005298	TRUE
159	0.235243	[0.08 0.38]	0.003115	CLU	0.048594	TRUE
159	0.244103	[0.09 0.39]	0.002134	FCGBP	0.03858	TRUE
159	0.361662	[0.22 0.49]	3.5E-06	FCN3	0.00041	TRUE
159	0.263949	[0.11 0.4]	0.00087	HABP2	0.020362	TRUE
159	0.243633	[0.09 0.39]	0.002178	HGFAC	0.03858	TRUE
159	0.253955	[0.1 0.4]	0.001379	SERPINF1	0.029337	TRUE
159	0.300807	[0.15 0.44]	0.000136	TGFBI	0.005298	TRUE
159	0.322168	[0.17 0.46]	4.11E-05	VTN	0.003203	TRUE

Supplementary Table 7: Proteins significantly correlated to steatosis grade from the SPEA plasma proteome, related to Figure 1.

n	r	CI95%	p-val	protein	pval_corrected	significant
159	0.283663	[0.13 0.42]	0.000333	AFM	0.026362	TRUE
159	0.30234	[0.15 0.44]	0.000125	AGT	0.013213	TRUE
159	0.313669	[0.16 0.45]	6.68E-05	DEFB1	0.010591	TRUE
159	0.33204	[0.18 0.46]	2.29E-05	PRAP1	0.007258	TRUE

Supplementary Table 9: Protein significantly correlated to NAS from the SPEA plasma proteome, related to Figure 1.

n	r	CI95%	p-val	protein	pval_corrected	significant
159	0.310327	[0.16 0.45]	8.06E-05	DEFB1	0.025552	TRUE

Supplementary Table 10: The MASH-regulated plasma proteins, related to Figure 1.

	**MASH v No Path**		**MASH v MASL**	
**Gene**	AdjPVal(-log10)	log2FC	AdjPVal (-log10)	log2FC
**TBCA**	2.371	1.899	1.642	2.469
**NDUFB6**	2.783	1.281	1.315	2.636
**AGT**	4.708	0.929	1.105	3.356
**APCS**	2.873	1.207	1.122	2.958
**RBP4**	2.857	1.181	1.366	2.231
**ORM1**	3.379	0.948	0.877	3.364
**PF4**	3.739	1.73	1.182	2.852
**ANG**	2.905	1.614	1.444	2.805
**HRG**	3.809	1.846	1.511	3.236
**SERPING1**	4.044	0.876	0.845	4.04
**IGKV4-1**	3.111	1.648	1.322	2.653
**TPM2**	2.395	2.371	2.389	3.015
**PF4V1**	3.41	1.648	1.065	2.52
**ORM2**	2.956	1.083	1	2.852
**SERPINA4**	3.197	1.073	0.998	3.078
**AFM**	4.416	0.929	0.739	3.759

Supplementary Table 13: MASH-regulated liver secreted proteins, related to Figure 2.

	**MASH v No pathology**		**MASH v MASL**	
**Gene**	**AdjPVal**	**log2FC**	**AdjPVal**	**log2FC**
VWA8	0.022	5.2	0.047	4.53
LPCAT2	0.043	3.07	0.045	3
CD63	0.012	2.97	0.015	2.82
GOLGA5	0	2.81	0.004	2.2
HBG1	0.017	2.2	0.042	1.9
FGA	0.019	1.95	0.032	1.84
FGG	0.017	1.93	0.043	1.66
PEX14	0.046	1.86	0.047	1.9
IGHA2	0.014	1.8	0.035	1.53
LBP	0.012	1.62	0.039	1.22
SELENOP	0.023	1.57	0.03	1.63
LRG1	0.04	1.47	0.047	1.37
LAMB2	0.015	1.11	0.045	0.88
CP	0.042	1.06	0.043	1.05
MRC2	0.017	1.01	0.045	0.85
LGALS1	0.039	0.98	0.043	0.95
PSMD10	0.012	0.97	0.002	1.05
PHYH	0.037	0.95	0.045	0.89
CTSH	0.017	0.94	0.024	0.91
NME2	0.017	0.67	0.032	0.59
MAPRE1	0.043	-0.48	0.043	-0.48
PSMC5	0.017	-0.67	0.039	-0.58
EIF3M	0.018	-0.88	0.039	-0.8
UPF1	0.022	-0.89	0.047	-0.77
UCHL5	0.017	-0.91	0.047	-0.73
NADSYN1	0.018	-0.97	0.039	-0.87
EIF4G3	0.047	-0.98	0.039	-1.08
VPS35	0.013	-1.06	0.017	-0.96
PRPF31	0.017	-1.16	0.004	-1.27
SPTA1	0.04	-1.32	0.03	-1.41
YTHDF3	0.014	-1.6	0.03	-1.44
SCLY	0.046	-1.69	0.049	-1.66
RELA	0.013	-1.91	0.012	-1.86
HECTD1	0.032	-2.35	0.045	-2.28
CRELD2	0.042	-4.5	0.039	-4.84
TUFM	0.017	-4.72	0.039	-4.28

Supplementary Table 16: Proteins significantly correlated to ballooning activity scores from the liver-secreted proteome, related to Figure 2.

**n**	**r**	**CI95%**	**p-val**	**protein**	**pval_corrected**	**significant**
96	0.382567	[0.19 0.54]	0.000154	CFI	0.046503821	TRUE
96	0.404762	[0.22 0.56]	5.72E-05	FGG	0.037503128	TRUE
96	-0.40333	[-0.56 -0.22]	6.11E-05	HSD17B2	0.037503128	TRUE
96	0.399056	[0.21 0.56]	7.43E-05	LRG1	0.037503128	TRUE
96	0.406033	[0.22 0.56]	5.39E-05	NME2	0.037503128	TRUE
96	0.396649	[0.21 0.56]	8.28E-05	ORM2	0.037503128	TRUE
96	0.392397	[0.21 0.55]	0.0001	PSMD10	0.037503128	TRUE
96	-0.37891	[-0.54 -0.19]	0.00018	SAMHD1	0.049869243	TRUE
96	-0.39026	[-0.55 -0.2]	0.00011	SEH1L	0.037503128	TRUE
96	0.389688	[0.2 0.55]	0.000113	SERPINF1	0.037503128	TRUE
96	-0.39631	[-0.56 -0.21]	8.41E-05	UPF1	0.037503128	TRUE
96	-0.41194	[-0.57 -0.23]	4.09E-05	YTHDF3	0.037503128	TRUE

Supplementary Table 17: Proteins significantly correlated to NAFLD activity score (NAS) from the liver-secreted proteome, related to Figure 2.

n	r	CI95%	p-val	protein	pval_corrected	significant
96	0.425613	[0.24 0.58]	2.11E-05	AGRN	0.01366	TRUE
96	0.443322	[0.26 0.59]	8.57E-06	CDHR2	0.007118	TRUE
96	0.53666	[0.37 0.67]	2.94E-08	FABP4	9.76E-05	TRUE
96	-0.44785	[-0.6 -0.27]	6.75E-06	GALE	0.007118	TRUE
96	-0.38909	[-0.55 -0.2]	0.000116	GCHFR.1	0.042776	TRUE
96	0.422415	[0.24 0.58]	2.47E-05	HRG	0.01366	TRUE
96	0.477266	[0.3 0.62]	1.32E-06	HSPG2	0.002189	TRUE
96	0.399848	[0.21 0.56]	7.16E-05	LAMB2	0.029734	TRUE
96	0.404362	[0.22 0.56]	5.82E-05	PLIN2	0.027626	TRUE
96	-0.38416	[-0.55 -0.2]	0.000144	PTGES3	0.047746	TRUE

Supplementary Table 18: Multivariate binary logistic regression and the predictive capacity of the covariates in the APASHA model to detect MASH in the discovery cohort, related to Figure 4.

**Variable**	**Coefficient (Z)**	**SE**	**P value**
Intercept	-13	2.861	<0.0001
AFM	2.547	0.812	0.0017
PCSK9	-1.085	0.4423	0.0141
APOF	-0.8873	0.4811	0.0651
S100A6	0.6684	0.251	0.0078
HbA1c	0.5208	0.2529	0.0394
AZGP1	1.239	0.4019	0.0021
**AUROC**	0.8875 (0.8200, 0.9550) (95% CI)	0.03444	<0.0001

Supplementary Table 19: Multivariate binary logistic regression and the predictive capacity of the covariates in the APASHA model to detect MASH in the discovery cohort, related to Figure 4.

**Cohort**	**MASH (n)**	**Model (Threshold)**	**AUROC (95% CI)**	**Sensitivity (n, %)**	**Specificity (n, %)**	**Positive predictive value (n, %)**	**Negative predictive value (n, %)**	**Likelihood ratio**
**Discovery (n=137)**	30	APASHA (>-1.217)	0.8875 (0.8200-0.9550)	(24/30) 80%	(96/117) 82.05%	(24/45) 46.66%	(96/102) 94.12%	4.457
**Validation (n=92)**	21	APASHA (>-1.217)	0.7673 (0.6644-0.8701)	(12/21) 57.14%	(52/71) 73.27%	(12/31) 38.71%	(52/61) 85.24%	2.135

AUROC – area under receiver operator characteristic curve; CI – confidence interval.

Supplementary Table 20: Z-score and significance of APASHA model compared to current non-invasive scores by Delong test, related to Figure 4.

	**Fib-4 score**		**Forn index**		**AST/ALT**		**CRP SPEA**		**TREM2**	
	Z	p value	Z	p-value	Z	p-value	Z	p-value	Z	p-value
**Discovery cohort**	5.1037	3.33E-07	5.1181	3.09E-07	4.309	1.64E-05	4.582	4.60E-06	3.7178	2.01E-04
**Validation cohort**	1.8845	0.0595	2.4672	0.01362	2.6646	0.00771	3.124	0.00178		

Supplementary Table 22: Simple non-invasive risk scores indices and equations, related to Supplementary Figure 1 & Figure 4.

Score	Indices	Calculation
FIB-4 Score[3]	Age, AST, ALT	Age × AST (U/L)/platelet count (×10^9/L) ×√ALT (U/L)
Forn index [4,5]	Platelets, GGT, age, cholesterol	7.811–3.131 × $\log_e$ (platelet [10^9/L]) + 0.781 × $\log_e$ (GGT [U/L]) + 3.467 × $\log_e$ (age [years]) − 0.014 × cholesterol [mg/dl]

ALT, alanine aminotransferase; AST, aspartate aminotransferase; BMI, body mass index; Fib-4, Fibrosis-4; GGT, gamma glutamyltransferase, T2D, type 2 diabetes; WCC, white cell count.

References:

1. Govaere, O., Hasoon, M., Alexander, L., Cockell, S., Tiniakos, D., Ekstedt, M., Schattenberg, J.M., Boursier, J., Bugianesi, E., Ratziu, V., et al. (2023). A proteo-transcriptomic map of non-alcoholic fatty liver disease signatures. Nature Metabolism *5*, 572-578. 10.1038/s42255-023-00775-1.
2. Li, Z., Zhang, H., Li, Q., Feng, W., Jia, X., Zhou, R., Huang, Y., Li, Y., Hu, Z., Hu, X., et al. (2023). GepLiver: an integrative liver expression atlas spanning developmental stages and liver disease phases. Scientific data *10*, 376. 10.1038/s41597-023-02257-1.
3. Subasi, C.F., Aykut, U.E., and Yilmaz, Y. (2015). Comparison of noninvasive scores for the detection of advanced fibrosis in patients with nonalcoholic fatty liver disease. Eur J Gastroenterol Hepatol *27*, 137-141. 10.1097/meg.0000000000000255.
4. Wu, S.-D., Wang, J.-Y., and Li, L. (2010). Staging of liver fibrosis in chronic hepatitis B patients with a composite predictive model: a comparative study. World journal of gastroenterology *16*, 501-507. 10.3748/wjg.v16.i4.501.
5. Ooi, G.J., Burton, P.R., Doyle, L., Wentworth, J.M., Bhathal, P.S., Sikaris, K., Cowley, M.A., Roberts, S.K., Kemp, W., O'Brien, P.E., and Brown, W.A. (2017). Modified thresholds for fibrosis risk scores in nonalcoholic fatty liver disease are necessary in the obese. Obes Surg *27*, 115-125. 10.1007/s11695-016-2246-5.
